# Contribution of Extracellular Vesicles in Rebuilding Injured Muscles

**DOI:** 10.3389/fphys.2019.00828

**Published:** 2019-07-18

**Authors:** Daniel C. Bittel, Jyoti K. Jaiswal

**Affiliations:** ^1^Children's National Health System, Center for Genetic Medicine Research, Washington, DC, United States; ^2^Department of Genomics and Precision Medicine, George Washington University School of Medicine and Health Sciences, Washington, DC, United States

**Keywords:** injury, exosomes, ectosomes, skeletal muscle, myogenesis, miRNA, endocytosis, ESCRT

## Abstract

Skeletal myofibers are injured due to mechanical stresses experienced during physical activity, or due to myofiber fragility caused by genetic diseases. The injured myofiber needs to be repaired or regenerated to restore the loss in muscle tissue function. Myofiber repair and regeneration requires coordinated action of various intercellular signaling factors—including proteins, inflammatory cytokines, miRNAs, and membrane lipids. It is increasingly being recognized release and transmission of these signaling factors involves extracellular vesicle (EV) released by myofibers and other cells in the injured muscle. Intercellular signaling by these EVs alters the phenotype of their target cells either by directly delivering the functional proteins and lipids or by modifying longer-term gene expression. These changes in the target cells activate downstream pathways involved in tissue homeostasis and repair. The EVs are heterogeneous with regards to their size, composition, cargo, location, as well as time-course of genesis and release. These differences impact on the subsequent repair and regeneration of injured skeletal muscles. This review focuses on how intracellular vesicle production, cargo packaging, and secretion by injured muscle, modulates specific reparative, and regenerative processes. Insights into the formation of these vesicles and their signaling properties offer new understandings of the orchestrated response necessary for optimal muscle repair and regeneration.

## Introduction

Skeletal muscle comprises over a third of the total human body mass, making it one of our largest organ systems. The contractile activity of the skeletal myofiber generates the force that enables and controls physical movement. This mechanical activity constantly subjects the skeletal muscle to stresses and strains that results in myofiber injury. Minor lesions inflicted upon skeletal myofibers, cause sarcolemmal disruption. The process of “myofiber repair” rectifies such membrane disruptions, preventing death of the injured myofiber. However, severe muscle injuries incurred via heavy overload, resistance training stresses, or genetic defects (e.g., muscular dystrophy), cause myofiber death. Such injuries are repaired by a highly orchestrated process involving inflammation and satellite cell activation and fusion that replaces the dead myofiber through the process of “myofiber regeneration.” Together, repair and regeneration of skeletal myofibers are essential for skeletal muscle maintenance in patients with chronic muscle disorders as well as restoration of functional performance in individuals that suffer sport injuries (Counsel and Breidahl, 2010; Tidball, 2017). Understanding the complexity of intercellular and intracellular interactions involved in myofiber repair and regeneration is essential for treating muscle diseases, and for the rehabilitation of individuals following muscle injury. Cellular and molecular events involved in repairing injured myofibers, and in regenerating damaged muscles, are extensively reviewed (Bentzinger et al., 2013; Yin et al., 2013; Andrews et al., 2014; Cooper and Mcneil, 2015; Horn and Jaiswal, 2018; Wosczyna and Rando, 2018). Here we will discuss how extracellular vesicle (EV)—membrane bound compartment released by cells, enable intracellular, and intercellular communication to coordinate repair and regeneration of the injured myofiber.

## Events That Facilitate Myofiber Repair and Regeneration

While myofiber repair relies on coordinated intracellular events, the process of myofiber regeneration involves coordinated intercellular interactions. The discussion below focuses on the role of vesicles and membrane trafficking processes that are involved in inter-and intra-cellular events that enable repair and regeneration of the injured skeletal myofibers.

### Myofiber Repair

**Figure 1:**
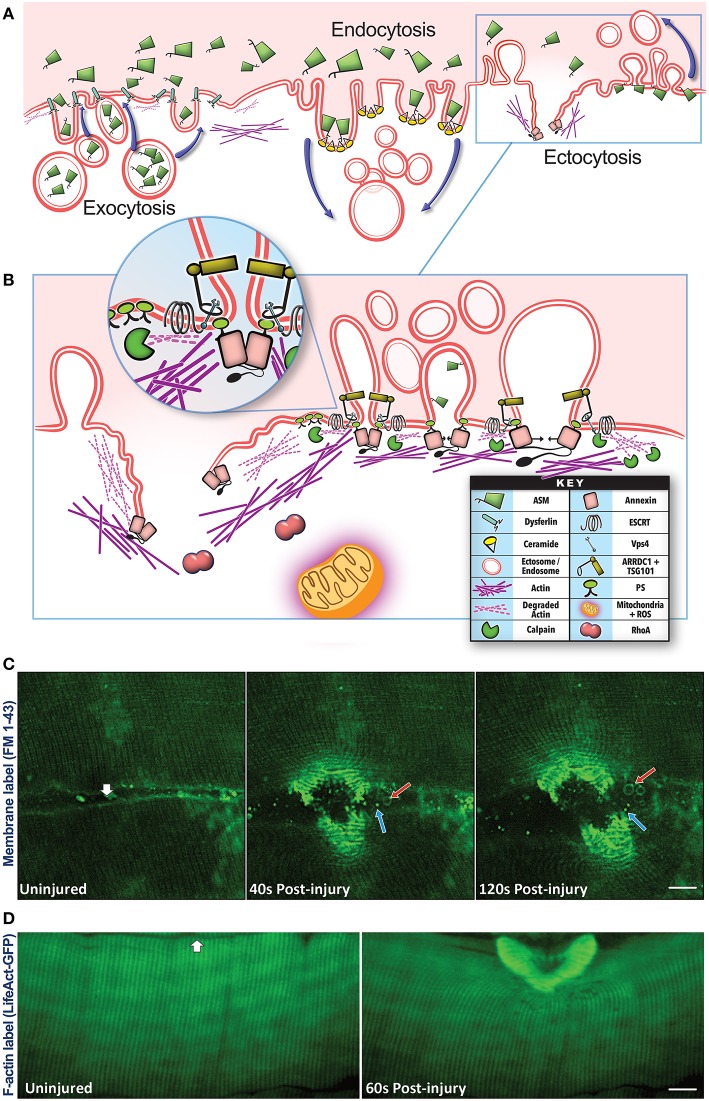
Vesicular pathways involved in plasma membrane repair. **(A)** Plasma membrane injury triggers membrane trafficking by Exocytosis—fusion of the intracellular vesicles such as lysosomes with the injured plasma membrane, Endocytosis—internalization of the plasma membrane, and Ectocytosis—shedding of the plasma membrane by way of microvesicles/ectosomes. Exocytosis is aided by calcium-binding membrane proteins such as dysferlin resulting in the release of lysosomal luminal proteins such as acid sphingomyelinase (ASM). The secreted ASM can access the outer and inner leaflets of the injured plasma membrane and hydrolyze the sphingomyelin lipids in these membranes to ceramide. Presence of ceramide in the outer leaflet will facilitate inward curvature and endocytosis, ceramide in the inner leaflet will cause outward curvature and ectocytosis. Both these processes enable removal of damaged membrane lipids from the site of injury by internalizing or shedding these lipids. **(B)** Ectocytosis is also facilitated by the interaction of proteins such as TSG101, ARRDC1, ESCRT III, and VPS4 as well as rearrangement of cortical actin beneath the membrane which help with vesicle budding and scission (*see inset*). Membrane shedding is also facilitated by the interaction of membrane lipids (phosphatidylserine) with the Annexin proteins and disassembly/reassembly of the cortical actin cytoskeleton with the help of calpain, Rho A, and Annexin proteins as well as mitochondrial ROS signaling. These latter processes also play a role in facilitating exocytosis and endocytosis indicating a complex set of membrane trafficking events that occur in concert, and failure, or delay in the any of these processes results in the failure of the injured myofiber to repair the plasma membrane injury. **(C)** Confocal images of live myofibers injured focally (white arrow) in the presence of membrane-impermeable FM 1–43 dye (green). Upon membrane injury, FM dye labels the intracellular membrane as well as all the vesicles secreted by the injured myofibers. Images of the same myofiber taken prior to and 40- or 120-s post injury show the formation of large (500–2,000 nm, red arrow) and small (<500 nm, blue arrow) extracellular vesicles. The released vesicles subsequently traffic away from the site of injury, but remain within the interfiber space (see Video 1). **(D)** Confocal images of a myofiber in an intact biceps muscle of Lifeact-GFP transgenic mouse showing F-actin response to focal injury (white arrow) by a 10 ms laser pulse. Images of the same fiber were taken just before and 60 s after injury, and show F-actin reorganization and buildup at the injury site.

**Figure 2:**
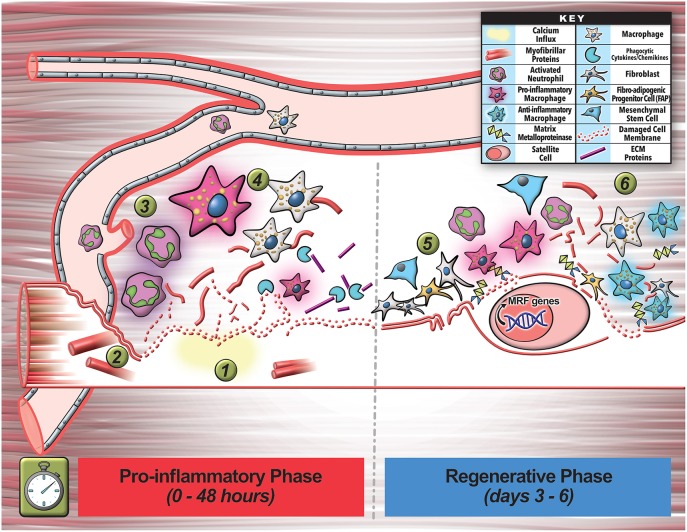
Multicellular interactions involved in early stages of myofiber regeneration. **(1)** Myofiber regeneration starts with the initial damage that causes calcium influx into the myofiber leading to release of damaged membrane, as well as breakdown and release of myofibrils and other cellular contents. **(2)** These and other factors released by the injured myofiber activates and recruits circulating immune cells (neutrophils) to the injury site to commence inflammation and initial phagocytosis of cellular debris. **(3)** Invading neutrophils secrete pro-inflammatory cytokines that promote inflammatory macrophage enrichment. **(4)** These cells help clear debris during initial inflammation and also secrete additional cytokines and chemokines that further assist in clearance of cell debris. **(5)** The inflammatory cells also signal proliferation of cells including the fibroblasts (FAPs) that assist in secretion of growth factors, cytokines, and extracellular matrix-remodeling enzymes (MMPs) that facilitate SC escape from the basal lamina and matrix remodeling required for regeneration. **(6)** In the final phase of the inflammatory response to injury, macrophages turn pro-regenerative, and together with the other additional cell types activate satellite cell (SC) myogenic program by activating regenerative transcription factors. This tightly orchestrated cellular choreography relies on intercellular communication via secretory factors including EVs that ultimately facilitates regeneration of the lost myofiber.

Plasma membrane damage results in a rapid influx of extracellular calcium, which triggers vesicular activity—internalization (endocytosis) and externalization (exocytosis, ectocytosis), and non-vesicular activity—local actin cytoskeleton reorganization to help repair the injury (Figures 1B,C). These activities help the myofiber repair, by sequestering the damaged part of the plasma membrane into endosomal vesicles or shedding it via EVs (Babiychuk et al., 2011; Keyel et al., 2011; Corrotte et al., 2013; Jimenez et al., 2014; Scheffer et al., 2014). Vesicle shedding can occur passively (Keyel et al., 2011; Boye et al., 2018) or through Endosomal Sorting Complexes Required for Transport (ESCRT)-mediated scission of the damaged membrane (Andrews et al., 2014; Jimenez et al., 2014; Scheffer et al., 2014; Demonbreun and Mcnally, 2017; Romero et al., 2017). Increase in intracellular calcium triggers exocytosis of vesicles such as lysosomes and late endosomes/multivesicular bodies (MVBs), by activating calcium binding proteins such as dysferlin and synaptotagmin. This allows the released vesicles to then accumulate locally and or distribute systemically (Figure 2).

**Figure 3:**
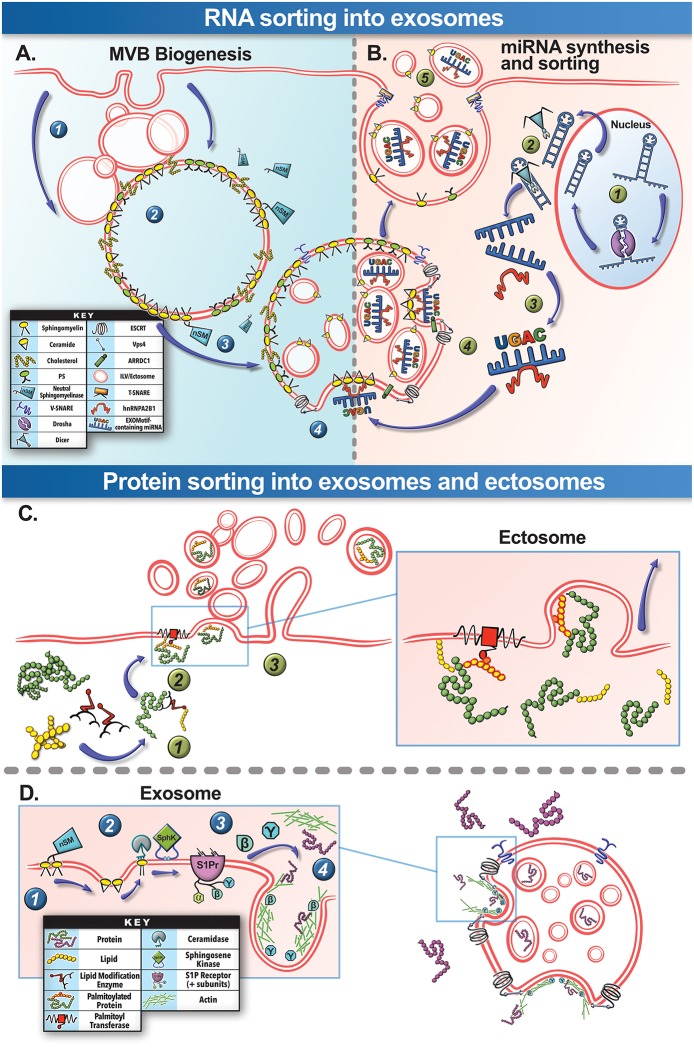
Biogenesis and selective cargo packaging into EVs. **(A)** MVB Biogenesis: Spontaneous or injury-triggered endocytosis of the plasma membrane forms **(1)** endosomes that **(2)** progress through the endosomal pathway to create the late endosome and the “multivesicular body” (MVB). The MVB membrane contains domains rich in sphingomyelin, cholesterol ceramide, and other specific lipids (lipid raft). **(3)** Cytosolic neutral sphingomyelinase (nSM), can hydrolyze sphingomyelin on the MVB membrane to generate ceramide that enables spontaneous inward budding of this region of the MVB membrane. **(4)** This inward budding is also assisted by the assembly of the ESCRT family proteins and is finally cleaved by Vps4, allowing the newly formed vesicles to be released into the MVB lumen as “intraluminal vesicles” (ILVs). **(B)** RNA Cargo Packaging: **(1)** Specific cellular stresses, such as cell injury, can trigger miRNA duplex transcription in the cell nucleus and then processed by Drosha. **(2)** This is followed by cleavage of the miRNA passenger strand by Dicer protein within the cytosol. **(3)** Based on the sequence of the miRNA, it may preferentially bind the cytosolic protein hnRNPA2B1. **(4)** These complexes can then associate the miRNA to ceramide at the nascent ILV and packaged into the ILV. **(5)** Subsequently these miRNA-containing ILVs are secreted as exosomes through the fusion of MVB via membrane-embedded v-SNARE proteins that interact with sarcolemmal t-SNARE proteins. This process helps exosomes functionally traffic miRNA to other cells and tissues. **(C)** Ectosome Protein Sorting: **(1)** Post-translational covalent lipid modification of the cysteine residues in the proteins is one of the mechanisms for protein sorting into ectosomes. This process is assisted by a family of lipid modification enzymes collectively known as acyltransferases. **(2)** Protein-membrane anchoring for stable ectosome packaging is achieved through palmitoylation by the membrane-bound palmitoyltransferase, anchoring the protein to the budding ectosome (*see inset for details*). **(3)** The protein containing ectosome can then be released by the cell. **(D)** Exosome Protein Sorting: For protein sorting into exosomes lipid signaling pathways on the MVB membrane play an important role. **(1)** Neutral sphingomyelinase hydrolyze sphingomyelin in the MVB membrane to ceramide. **(2)** Ceramide is subsequently catabolized to sphingosine and sphingosine-1-phosphate (S1P) by cytosolic ceramidase and sphingosine kinase (SphK), respectively. **(3)** S1P continuously activates S1P receptors on the MVB membrane, stimulating the release of their β and γ subunits. **(4)** This catalyzes GTPases and actin-mediated sorting pathways for loading proteins into the budding ILVs/exosomes. A parallel pathway not depicted here involves ESCRT complex-mediated sorting and packaging of ubiquitinated proteins on the MVB membrane into ILVs for subsequent degradation or secretion.

Lysosome exocytosis contributes endomembrane to help close the wound, and also enables release of the lysosomal enzyme acid sphingomyelinase (ASM) (Chakrabarti et al., 2003; Jaiswal et al., 2004; Defour et al., 2014; Sreetama et al., 2016; Figure 1A). Upon its release ASM gains access to the plasma membrane lipids including sphingomyelin, and hydrolyzes it to generate ceramide—a lipid that is enriched in injured cells (Babiychuk and Draeger, 2000; Tam et al., 2010; Corrotte et al., 2013; Romancino et al., 2013; Figure 1A). This ceramide facilitates plasma membrane repair by removing the damaged membrane through internalization (via endosomes) and shedding (via ectosomes) (Draeger and Babiychuk, 2013; Figures 1A–C). ASM inhibitors and ASM deficiency blocks membrane removal (Bianco et al., 2009) and membrane repair (Corrotte et al., 2013; Deng et al., 2017; Michailowsky et al., 2019). The endocytosed vesicles can progress through the endosomal pathway, whereby inward budding of the injured membrane forms ~300–500 nm-sized intracellular endocytic vesicles that fuse together to form late endosomes and MVBs in the injured cells (Murphy et al., 2018; Figures 1A, 3A). These endosomes may degrade the internalized damaged proteins and lipids, or undergo inward budding to create intraluminal vesicles (ILV) (Murphy et al., 2018). The MVBs can then traffic and fuse to the membrane to exocytose their contents. These ILVs are subsequently released from the cell upon MVB exocytosis, and these released vesicles are called “exosomes” (Figures 1C, 3B). Through these vesicle trafficking events, the injured plasma membrane is not only repaired within minutes of injury, but the EVs generated and released in the process of repair can subsequently signal extracellularly to affect a tissue-level repair response that can continue beyond the brief myofiber repair phase (see Figures 1, 2).

For the above vesicular events to remodel damaged membranes, cells rely on cytoskeletal proteins to stabilize the damaged membrane and assist in ferrying vesicles to and from the damaged membrane (Mcdade et al., 2014; Vaughan et al., 2014; Horn and Jaiswal, 2018; Figure 1D). Calcium influx due to cell membrane disruption activates kinases and lipid modifying enzymes, creating a signaling platform that assists with vesicle fusion, lipid, and actin reorganization, and constriction of membrane wound during repair (Floyd et al., 2001; Mandato and Bement, 2001; Benink and Bement, 2005; Vaughan et al., 2014; Horn and Jaiswal, 2018). Injury-induced calcium entry also activates Calpains, which help disassemble the cortical actin cytoskeleton allowing intracellular vesicles to access the cell membrane and repair the membrane by fusion. This calpain-mediated actin loss also relieves membrane tension, preventing cytoskeletal contraction from pulling the lipids in the damaged free-edges of the wounded membrane outward, which would cause the wound to expand (Gitler and Spira, 1998; Togo et al., 2000; Mcneil et al., 2001; Hendricks and Shi, 2014; Redpath et al., 2014). Moreover, lipids in the wounded membrane facilitate binding of specific proteins such as annexins. Annexin family proteins subsequently assist in closing the wound by curving the membrane wound edges via their construction of 2-dimensional proteins arrays to stabilize the membrane, and by stimulating actin reorganization and membrane scission (Bouter et al., 2011; Boye et al., 2017; Figures 1B,D). Another signal that regulates reorganization of actin and cytoskeletal proteins is reactive oxygen species (ROS) produced by mitochondria. However ROS is also produced during-, and has an additional role in-, membrane repair (Cai et al., 2009; Spaeth et al., 2012; Duan et al., 2015; Horn et al., 2017; Figure 1B). Restoration of membrane structure and function is a key early feature in successful repair and regeneration of skeletal muscle, failure of which leads to myofiber degeneration, requiring myofiber regeneration through intracellular signaling (Tidball, 2011; Demonbreun et al., 2016). However, even myofibers that successfully repair release signals in the form of secreted molecules and vesicles that informs the tissues of the muscle damage. Thus, a continuum of extracellular responses triggered by damaged, repaired, regenerating, and degenerating myofibers, facilitate the intracellular signaling following membrane injury that supports healthy regeneration and growth of the injured muscle.

### Myofiber Regeneration

Similar to the complexity of myofiber repair, myofiber regeneration also depends on a series of highly coordinated events, orchestrated between multiple cell types that engage in intercellular communication (Chazaud, 2016; Wosczyna and Rando, 2018). Inadequate membrane repair initiates myofiber necrosis, triggering an inflammatory response, and subsequently culminating in regeneration of the lost myofiber by activation of muscle-resident stem cells satellite cells (SCs). Dysfunction in this process results in myofiber loss and replacement by fibrosis, adipogenesis, or calcification, preventing structural, and functional restoration of the injured muscle (Wosczyna and Rando, 2018). Unlike myofiber repair, where local/mild activation of calpain helps restore myofiber integrity, excessive calcium influx activates calpain-mediated loss of myofibrils and extracellular exposure of phosphatidylserine (PS) membrane lipids (Figure 2). While myofibril damage causes myofibers to degenerate, PS exposure is one of many signals that activate the recruitment of circulating immune cells to initiate the inflammatory response and initial phagocytosis of myofibrillar debris (Demonbreun and Mcnally, 2017; Lemke, 2019). Amongst the first responders to muscle injury are neutrophils that rapidly invade the damaged tissue within hours, reaching their peak at 24–48 h after muscle injury (Bentzinger et al., 2013; Demonbreun and Mcnally, 2017; Tidball, 2017; Figure 2). These neutrophils signal to enrich the muscle environment with pro-inflammatory cytokines, activating a pro-inflammatory response by macrophages (Tidball, 2017). These pro-inflammatory macrophages help with phagocytic clearance of debris and stimulate SCs to proliferate and proceed down the myogenic program (Figure 2). This initial inflammatory response also involves other cell types including regulatory T-cells, fibroblasts, and fibro-adipogenic-progenitors (FAPs) that assist in removal and cleanup of damaged cells and proteins over the course of 3–4 days post-injury—processes needed for proper extracellular matrix (ECM) turnover and subsequent regeneration of functional muscle tissue (Tidball, 2017; Wosczyna and Rando, 2018; Figure 2). These immune cells secrete a wealth of diffusible factors, such as growth factors, inflammatory cytokines (IL-6), globular adiponectin, ECM components, and ECM-remodeling matrix-metalloproteinases (MMPs) that not only generate ECM chemoattractive fragments, but help SCs escape from the basal lamina to engage in regeneration (Bentzinger et al., 2013; Chazaud, 2016; Figure 2).

In the second phase of the inflammatory response to muscle injury—around 4 days post-injury, macrophages shift to a pro-regenerative phenotype and secrete anti-inflammatory cytokines and growth factors to facilitate SC proliferation and differentiation leading to their fusion and formation of nascent myofibers (Bentzinger et al., 2013; Chazaud, 2016; Figure 2). This regenerative and remodeling phase also hinges upon intercellular coordination and communication involving immune cells, SCs, FAPs, pericytes, fibroblasts, and endothelial cells that secrete proteins and molecules influencing neighboring cells, notably culminating in SC differentiation (Bentzinger et al., 2013; Wosczyna and Rando, 2018; Figure 2). This stimulated myogenic differentiation program is an irreversible process driven by the sequential expression of transcription factors myogenic regulatory factors (MRFs) that induce gene expression signals to target genes within the SCs. A large proportion of these MRF target genes largely encode muscle-specific structural and contractile proteins (actins, myosins, and troponins) essential for formation of functional skeletal muscle (Bentzinger et al., 2013). However, other cofactors such as miRNAs (e.g., miR-1, miR-133, miR-206) also influence myogenic differentiation through modulation of MRF levels as well (Bentzinger et al., 2013).

It has become evident that muscle repair and regeneration requires coordinate expression of various factors including secreted proteins, inflammatory cytokines, miRNAs, and membrane lipids that transfer intercellular signals (Murphy et al., 2018). However, an increasingly recognized means of intercellular signaling in muscle repair and regeneration involves release and uptake of vesicles (Demonbreun and Mcnally, 2017). A recent study in a murine model, examining the effects of acute muscle injury due to a 20 min downhill running exercise, demonstrated increased circulating vesicles, both during the initial hours after myofiber injury and during subsequent regeneration 5–7 days post-exercise damage (Coenen-Stass et al., 2016). This suggests that the vesicles that carry signals during regeneration are produced by the injured myofiber and regenerative cells alike, and that these may play distinct but complimentary roles in the repair response to injury (Coenen-Stass et al., 2016). Insights into the formation of these vesicles and their signaling properties may reveal new understandings of the orchestrated response necessary for proper muscle repair and regeneration.

## Extracellular Vesicles—Exosomes and Ectosomes

Similar to other cells, skeletal myofibers broadly produce 3 types of extracellular vesicles—apoptotic bodies, exosomes, and ectosomes. These vesicles are distinguished by their size, composition, and cellular origin. The largest of these vesicles—apoptotic bodies (1,000–8,000 nm diameter), derive from apoptotic tissues in which cell repair and regeneration has failed (Crescitelli et al., 2013; Silva et al., 2017; Caruso and Poon, 2018). Hence, we will focus on the other two groups of EVs—exosomes and ectosomes (microvesicles). Exosomes are smaller in size, with diameter ranging from ~30–150 nm, while ectosomes range from ~150–1,000 nm (Crescitelli et al., 2013; Villarroya-Beltri et al., 2013; Janas et al., 2015; Leoni et al., 2015; Choi et al., 2016; Wang and Wang, 2016; Demonbreun and Mcnally, 2017; Meldolesi, 2018; Murphy et al., 2018). Aside from their size however, these vesicles fundamentally differ in their composition and the cellular compartments from which they are manufactured (Table 1).

**Table 1:**
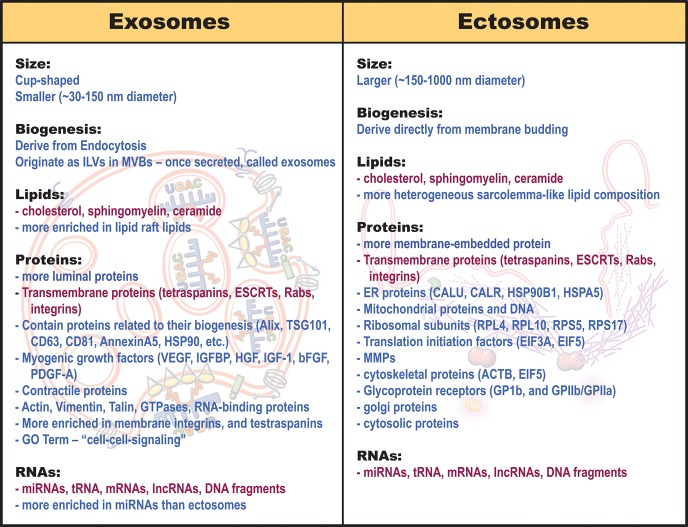
Properties of muscle derived exosomes and ectosomes.

*Exosomes and Ectosomes share many common features (red text), but also present a number of unique features and cargoes (blue text)*.

Exosomes, also known as “intraluminal vesicles” (ILVs), develop from a sequential process of MVB membrane remodeling (Figure 3A). The MVB membrane contains lipid domains not unlike the muscle sarcolemma from which they are derived (Murphy et al., 2018; Figure 3A). Lipid-protein interactions and lipid reorganization at MVB membrane domains enables spontaneous inward budding of these regions, that is further aided by ESCRT-family proteins and released into the MVB lumen by Vps4 ATPase-mediated cleavage as ILVs (Trajkovic et al., 2008; Colombo et al., 2014; Cocucci and Meldolesi, 2015; Janas et al., 2015). The ILVs are thus enriched in the structural lipids of the MVB membrane that help their formation (Janas et al., 2015). The MVBs subsequently traffic to the plasma membrane where SNARE proteins and tethering factors such as SNAP23, Syntaxin1a, VAMP7 and 8, Rabs 11, 27, and 35 aid in MVB exocytosis, releasing ILVs as exosomes (Colombo et al., 2014; Cocucci and Meldolesi, 2015; Janas et al., 2015; Meldolesi, 2018; Figures 3A,B).

Ectosomes, are produced at the plasma membrane by a different cellular process that involves small GTPases Arf6, Rab22, RhoA, CDC42, and Rac1 and the contractile activity of cortical actin beneath the cell membrane (Muralidharan-Chari et al., 2009; Wang et al., 2014; Meldolesi, 2018). However, ectosomes can also be formed using some of the same cellular machinery (e.g., ESCRTs) used by exosomes (Muralidharan-Chari et al., 2009; Scheffer et al., 2014; Cocucci and Meldolesi, 2015; Meldolesi, 2018; Figure 1). A proteomic screen in muscle cells identified several ESCRT III proteins (Chmps 1, 4, and 6) and only one ESCRT I protein (Tsg101) accumulates at the site of muscle cell membrane injury (Scheffer et al., 2014). TSG101 assists in ectosome formation by interacting at the ectosome neck with the arrestin domain-containing protein-1 (ARRDC1) and with ESCRT III/ Vps4 complex to enable pinching and release of the ectosome (Nabhan et al., 2012; Dobro et al., 2013; Scheffer et al., 2014; Figure 1B).

## Functionalizing the Extracellular Vesicles by Active and Selective Cargo Loading

The differences in cellular origin of exosomes and ectosomes lead to differences in their structural components and the cargoes they carry (Cocucci and Meldolesi, 2015; Mcguinness et al., 2016; Silva et al., 2017; Taverna et al., 2017). Lipid, protein and RNA cargo composition in these vesicles are distinct, not only from that of the cytoplasm but also between exosomes and ectosomes (Table 1). This indicates that cargo packaging in the vesicles is an active and selective process that may be responsive to the cell's physiology (Roberts et al., 2012; Matsuzaka et al., 2016; Panagiotou et al., 2016; Fry et al., 2017; D'souza et al., 2018). However, despite these differences, these vesicle subtypes share a range of common lipids, transmembrane proteins, surface ligands, and cargoes (Mcguinness et al., 2016; Silva et al., 2017; Taverna et al., 2017; Table 1, red text).

### Protein and Lipid Cargo

Due to their generation from the raft-like region of the MVB membrane, exosomes contain lipids that are enriched in the MVB lipid raft domains, as discussed above (Janas et al., 2015). Conversely, being derived from the plasma membrane, ectosomes contain a more heterogeneous plasma membrane-like lipid composition (Meldolesi, 2018; Table 1). Moreover, vesicle differences extend beyond lipid composition, with as much as 65% of protein cargoes reported to differ between muscle exosomes and ectosomes (Le Bihan et al., 2012). This highlights the divergent protein sorting pathways employed by the vesicle generating cell to control vesicle cargoes and consequentially, their signaling properties after muscle damage (Laterza et al., 2009; Roberts et al., 2012).

Proteomic analysis of muscle-derived exosomes shows that in addition to proteins involved in their biogenesis, the exosomes also contain functionally important proteins such as myogenic growth factors and contractile proteins (Choi et al., 2016; Demonbreun and Mcnally, 2017; Table 1). Compared to ectosomes, which are enriched for membrane-embedded proteins, exosomes are enriched in proteins including integrins, MHC molecules, tetraspanins, ESCRTs, endosome proteins, and even zinc-finger transcription factors (Le Bihan et al., 2012; Tables 1, 2). Gene-ontology analysis of muscle exosome proteins show that they largely belong to the protein families involved in “cell-cell signaling” (Le Bihan et al., 2012). Unlike their exosome counterparts, due to their immediate generation at the muscle sarcolemma, ectosomes, contain more transmembrane proteins, receptors, glycoproteins and metalloproteinases (MMPs) (Meldolesi, 2018; Table 1). They are also enriched in proteins normally found in the endoplasmic reticulum, mitochondria (dehydrogenase proteins, respiratory electron transport chain), golgi, cytoskeleton, and cytosol (Le Bihan et al., 2012; Kowal et al., 2014; Phinney et al., 2015; Willms et al., 2016; Meldolesi, 2018; Tables 1, 2). Gene ontology analysis of muscle ectosome proteins shows predominance of proteins involved in RNA post-translational modification, amino acid metabolism, protein synthesis, molecular transport, and protein degradation (Le Bihan et al., 2012). Thus, there are notable differences in the protein and membrane lipid composition of exosomes and ectosomes, which in-turn influences uptake and function of these EVs in the target cells (see the section Extracellular Vesicle Uptake by the Target Cells for details). However, aside from cellular compartment location, cell sorting pathways may influence the differential cargo packaging in these vesicles as well.

**Table 2:** Proteins enriched in skeletal muscle exosome and ectosome fractions.

**Proteins enriched in Ectosomes**	**SwissPalm palmitoylation site**	**Cellular role (skeletal muscle)**
**Endoplasmic reticulum proteins**		
CALU, HSP90	CALU—Cysteine 9, 14 HSP90—Cysteine 420	ER calcium-binding protein (protein folding/sorting) HSP90-induces inflammation, activates TLRs on macrophages and muscle cells to stimulate muscle catabolism (Zhang et al., 2017)
**TCP-1 chaperonins** TCP-1	Cysteine 147	Key protein in actin biogenesis at z-disc during sarcomere assembly (Berger et al., 2018)
**Actins and tubulins** ACTB, TUBA1B	ACTB—Cysteine 17 TUBA1B—Cysteine 376	TUBA1B-trafficking of dysferlin to the sarcolemma in membrane repair (Azakir et al., 2010)
**Ribosomal subunits** RPL4, RPL10, RPS5, RPS17	RPL4—Cysteine 3 RPL10—Cysteine 80 RPS5—Cysteine 172, 66 RPS17—Cysteine 35	All involved in ribosomal structure and protein translation in the cytosol for cell growth (Bennet et al., 2018)
**Translational initiation Factors** EIF5	Cysteine−22, 38	Crucial for satellite cell differentiation – delivers tRNA to ribosome to initiate protein translation (Luchessi et al., 2009; Jennings and Pavitt, 2010)
**Matrix metalloproteinases** MT1-MMP	Cysteine−574	Required for fibronectin degradation and basement membrane laminin cleavage for proper elongation and myoblast fusion (Ohtake et al., 2006)
**Glycoprotein receptors** GPIb	Cysteine−352	Involved in gluconeogenesis (Lamanna et al., 2015)
**Adhesion proteins** P-selectin	Cysteine−807	Required for leukocyte recruitment to injured skeletal muscle (Frenette et al., 2003)
**Integrin** Mac-1	Cysteine 14	Required for leukocyte recruitment after skeletal muscle injury (Lagrota-Candido et al., 2010)
**Proteins enriched in Exosomes**	**SwissPalm palmitoylation site**	**Cellular role (skeletal muscle)**
**Intermediate Filament (z-disc)** Vimentin	Cysteine−328	Required for structural remodeling of skeletal muscle, especially at myotendinous junction (Vaittinen et al., 2001)
**Cytoskeletal protein** Talin	NA	Required for myoblast fusion, sarcomere assembly, and myotendinous junction maintenance (Conti et al., 2009)
**Annexins** ANX-A1, ANX-A4, ANX-A6	ANXA1—Cysteine 343, 263 ANXA6—Cysteine 114	Anti-inflammatory signaling, signal transduction, cytoskeleton and ECM integrity, muscle growth, satellite cell differentiation, migration, and fusion, and muscle membrane repair (Bizzarro et al., 2012a,b)
**GTPases** ARF-6	NA	Regulator of myoblast fusion via phospholipase D activation (triggers phospholipid production and actin reorganization at myoblast fusion sites) (Bach et al., 2010)
**RNA-binding proteins** Argonaute 2, Y-box1	NA	AGO2—facilitates packaging of miRNAs into exosomes, protects miRNA from lysosomal degradation, required for miRNA silencing of mRNA translation (Lv et al., 2014)
		Y-box1—nucleic acid chaperone involved in DNA replication and repair, transcription, pre-mRNA splicing and mRNA translation, particularly of genes involved in cell division, apoptosis, immune response (Eliseeva et al., 2011)
**Integrins** ITGA4, ITGA6, ITGA7	ITG4—Cysteine 25 ITGA6—Cysteine 8, 26	Integrins—serve as mechanotransducers connecting ECM to cell cytoskeleton to influence signaling cascades involved in myogenesis, hypertrophy (Carson and Wei, 2000). Also play role in myoblast migration and fusion (Mayer, 2003)
**MHC molecules** HLA-A, HLA-B	NA	HLA-A and B—MHC class 1 cell surface receptors that present peptides recognized by inflammatory T-cells leading to inflammation and apoptosis (Appleyard et al., 1985)
**Endosomes** TSG101	NA	TSG101—Governs ILV and exosome formation in the late endosome/MVB (Edgar, 2016)
**Lysosome** LAMP2	NA	LAMP2—governs protein sorting into degradative lysosomes (autophagasomes) (Zhang et al., 2017)

*The palmitoylated cysteines are as reported in SwissPalm [weblink]. All of the validated ectosome-enriched proteins appear to contain the palmitoylation sites, while only a few exosome proteins possess these cysteine residues. The roles reported for these cargoes include roles in the health and maintenance of skeletal muscle, as well as repair and regeneration of muscle following injury*.

Aside from the cellular location where the EVs are produced, active protein sorting pathways also influence the cargo packaging in these vesicles (Yang and Gould, 2013). One example of this is “curvature-based sorting” (Hanson et al., 2009; Yáñez-Mó et al., 2009, 2015; Nazarenko et al., 2010; Perez-Hernandez et al., 2013). Protein intrinsic molecular shape can drive its diffusion and membrane routing to flat membrane spaces or curved, vesicle-generating areas, as this limits membrane free energy, while proteins with curvature-favoring structural elements (i.e., BAR domains), can actually drive the formation of vesicles themselves (Arkhipov et al., 2008). For instance, in ectosome formation, ESCRT complex proteins energetically favor the neck region of the budding ectosome, while tetraspanins tend to form tetraspanin-enriched microdomains that help drive ILV budding on the MVB membrane (Hanson et al., 2009; Yáñez-Mó et al., 2009). Such protein sorting mechanisms may partially account for the frequent findings of ESCRT proteins within ectosome vesicles and tetraspanins within exosomes (Nazarenko et al., 2010; Perez-Hernandez et al., 2013). Of particular relevance, BAR domain proteins, such as BIN-3, may be required for muscle regeneration due to its ability to promote satellite cell fusion and migration via lamellipodia formation (Simionescu-Bankston et al., 2013). However, further studies are warranted to investigate if BAR-domain proteins in EVs play a role in muscle regeneration.

Assembly of cytosolic cargo proteins into the lumen of ectosomes requires binding of these proteins to the plasma membrane—a process reliant on post-translational protein lipid-modification (Yang and Gould, 2013; Figure 3C). These modifications include covalent lipid attachment on the cysteine side chain of myristoyl (myristoylation), isoprenoid (prenylation), or palmitoyl (palmitoylation) moieties (Aicart-Ramos et al., 2011). These modifications can attract and sustain/anchor the tagged cytosolic proteins from their N- or C-terminal ends to the ectosome budding site on the membrane (Yang and Gould, 2013; Cocucci and Meldolesi, 2015; Figure 3C). This process is dynamic, highly regulated, reversible, and governed by a family of enzymes e.g., DHHC palmitoyl transferases, deacylases, and prenyltransferases (Aicart-Ramos et al., 2011; Cocucci and Meldolesi, 2015). Interestingly, these protein anchors are not efficient in protein targeting for sorting into exosomes (Yang and Gould, 2013). Examination of skeletal muscle cell ectosome proteome reveals an abundance of proteins with predicted palmitoylated cysteine residues, while exosome-enriched proteins do not possess such anchoring/modification sites (Blanc et al., 2015; Table 2). In this respect, protein lipid modifications within the cell may serve as a key mechanism of selective cargo loading to influence intercellular signals produced by skeletal muscle in the repair and regeneration process. It is worth noting that following burn injury, skeletal muscle increases expression of prenyltransferase and of protein prenylation, inhibition of which reduces inflammatory gene expression in skeletal muscle, implicating protein prenylation in inflammation following skeletal muscle injury (Nakazawa et al., 2015). Moreover, tissue regeneration-specific protein palmitoylation has been shown to be critical for neural scaffolding and dendritic spine maturation in neuronal regeneration (Zhang and Hang, 2017). Therefore, given the connection between protein acylation and ectosome packaging and its role in tissue inflammation and regeneration, probing the role of protein lipid modification in vesicle-mediated intercellular signaling after skeletal muscle injury is an interesting area for future investigation.

Aside from protein-lipid modification and membrane anchoring in cargo sorting into ectosomes, other intracellular lipid signaling processes have also emerged as means of protein cargo sorting into exosomes (Kajimoto et al., 2013, 2018; Janas et al., 2015). For example a chain of sequential lipid intracellular lipid modifications at the MVB membrane, concluding with S1P receptor activation and Rho-family GTPAse stimulation, acts as a signaling axis for protein sorting into exosomes (Kajimoto et al., 2013, 2018; Figure 3D). Aberrant lipid hydrolysis, SphK activity/phosphorylation status, and S1P subunit release, alters exosome protein cargo packaging and muscle repair (Saba and De La Garza-Rodea, 2013; Guo et al., 2015; Janas et al., 2015; Badawy et al., 2018; Kajimoto et al., 2018). In skeletal muscles, eccentric-contraction-induced injury activates SphK1 and increases endogenous S1P synthesis, while selective inhibition of SphK1 during eccentric injury promotes muscle fibrosis, attenuates extracellular matrix remodeling, and exacerbates myofiber damage (Sassoli et al., 2011; Loh et al., 2012). Further, increasing skeletal muscle S1P levels improves muscle regeneration in a mouse model of Duchenne muscular dystrophy (DMD), while also increasing myofiber size, force, and SC abundance, and diminishing fibrosis and adipose tissue accrual (Ieronimakis et al., 2013). Given the impact of altered S1P signaling in exosome protein sorting and in skeletal muscle regeneration, there is a need to determine if altered exosome packaging due to aberrant S1P activity contributes to poor reparative response to muscle injury (Donati et al., 2013).

### RNA Cargo

EVs differ from their parent cell in RNA composition—unlike the abundance of rRNA in the parent cell, EVs are comparatively enriched in small RNAs such as miRNAs (Crescitelli et al., 2013; Jeppesen et al., 2019; Table 1). Further, similar to their differential protein contents, exosomes and ectosomes have different RNA cargoes (Jeppesen et al., 2019; Table 1). RNA-seq analysis of EV subpopulations identified that of the RNA transcripts unique to EVs, ectosomes contain only ~9–14% of the vesicle-enriched RNA species (Chen et al., 2016). This is attributed to differential RNA sorting mechanism—miRNAs are preferentially sorted into exosomes based upon their unique nucleotide sequences (Roberts et al., 2012; Matsuzaka et al., 2016; Fry et al., 2017; D'souza et al., 2018; Table 3). In muscle cells damaged by eccentric exercise or due to muscular dystrophy, tissue-enriched miRNAs (myoMirs) are seen to be elevated in the circulation, specifically during the regenerative phase *in-vivo* and during myoblast differentiation *in-vitro* (Coenen-Stass et al., 2016). However, after muscle damage, the level of many of these miRNAs declines within muscle and are detected only in vesicles, suggesting their selective packaging and release, as opposed to passive leak, in response to muscle damage (Siracusa et al., 2016). Moreover, vesicle-associated myomiRs differ between dystrophy-associated damage and eccentric muscle damage, further suggesting a context-specificity of miRNA loading into the exosomes (Roberts et al., 2012; Matsuzaka et al., 2016; Fry et al., 2017; D'souza et al., 2018).

**Table 3:** miRNA EXOmotif: commonly reported muscle-damage induced miRNAs.

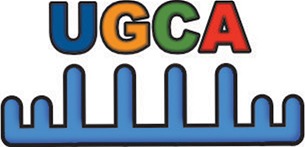	NA	CLmotif—sorts the miRNA into the cell (not packaged in exosomes). Not demonstrated within commonly reported muscle-damage induced miRNAs.
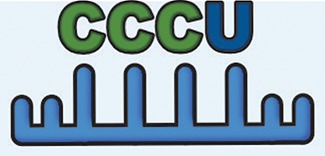	miR-133B miR-486	Involved in SC proliferation, differentiation, fiber-type specification, exercise adaptation (Horak et al., 2016; Siracusa et al., 2016; Jin et al., 2017) represses Pten and FoxO-1a to reinforce Akt signaling and enhance muscle growth (Small et al., 2010)
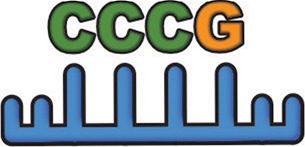	miR-206	Promotes myoblast differentiation and promotes terminal maturation of muscle fibers (Kim et al., 2006; Windbanks et al., 2013; Siracusa et al., 2016)
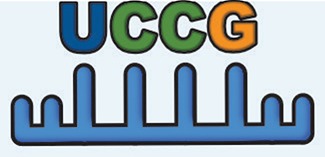	miR-27B miR-14	Increases proliferation of myogenic cells (Ling et al., 2018) positively regulates myogenin and MyHC by targeting the epigenetic regulator Ezh-2 (member of the polycomb repressive complex) (Juan et al., 2009)
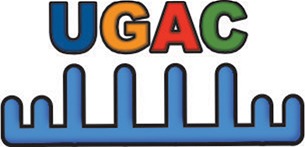	miR-149	Released and peak in circulation 1 h and 1 day after resistance exercise. Its role is unclear (Sawada et al., 2013)
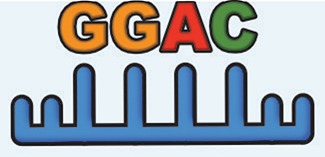	miR-1	miR-1 promotes differentiation of myoblasts via repression of Hdac-4 that represses Mef-2-depednent expression of myogenic factors (Chen et al., 2010; Di Filippo et al., 2016; Siracusa et al., 2016)
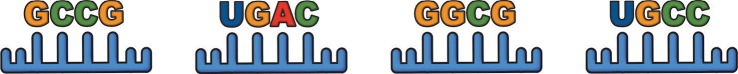		

*With the exception of the CLMotif UGCA, each EXOMotif is found in miRNA's reported to be elevated in skeletal muscle exosomes following injury. The reported roles of these miRNAs in repair and regeneration of skeletal muscle include pro-myogenic effects on muscle satellite cells and activation of myogenic transcription factors*.

Simultaneous with miRNA transcription and cleavage within the cell cytosol, these cytosolic miRNAs bind the outer MVB membrane to be packaged into nascent ILVs as they bud inwards (Janas et al., 2015; Figure 3B). This sorting of these RNAs into the ILVs is based on affinity of the RNAs to the MVB raft regions, which is dependent upon the RNAs nucleotide-sequence termed EXOmotif (Villarroya-Beltri et al., 2013). Thus, the presence of these motif(s) in miRNA preferentially sorts them into exosomes, and mutating these EXOmotifs, prevents their import into exosomes, retaining them in the cytoplasm (Villarroya-Beltri et al., 2013). There are currently 10 annotated EXOmotifs responsible for sorting of the ~75% of known miRNA found in exosomes, and then there are established CLmotifs that predisposes miRNA retention within the cytosol (~67% of the time) (Janas and Janas, 2011; Janas et al., 2012; Villarroya-Beltri et al., 2013; Table 3). EXOmotifs can dictate RNA binding to- and attraction of- cytoskeleton-associated cytosolic proteins (hnRNPA2B1, Y-box protein 1, Major Vault Protein) that assist in miRNA shuttling to the MVB (Villarroya-Beltri et al., 2013; Santangelo et al., 2016), a process that confers specificity to exosome miRNA packaging (Villarroya-Beltri et al., 2013; Figure 3B). Lack of these proteins reduces miRNA sorting into exosomes (Shurtleff et al., 2016; Teng et al., 2017; Statello et al., 2018). The evidence for specific sorting of miRNAs into exosomes following muscle damaging stimuli, comes from studies examining exosome cargoes. Following a bout of muscle damaging cycling, exosomal release of EXOmotif containing miRNAs—miR-208a, miR-126, and miR-16 increases (D'souza et al., 2018). Similarly, overload-induced muscle damage causes release of the CCCG-EXOmotif containing miR-206 within SC-derived exosomes (Fry et al., 2017). Conversely, DMD-associated muscle damage promotes exosomal release of GGAC-EXOmotif-containing miR-1, CCCG-containing miR-206 (Roberts et al., 2012), and CCCU containing miR-133a (Matsuzaka et al., 2016). With the importance of these miRNAs in intercellular signaling for regeneration, their selective packaging and sorting after muscle damage may be a purposeful regulatory mechanism (Table 3).

## Extracellular Vesicle Uptake by the Target Cells

In order to transmit their signals, both the EVs find and interact similarly with their target cell to deliver their cargoes into the host cell cytosol, this includes direct fusion with the target cell membrane, or internalization via endocytosis (Rejman et al., 2004; Cocucci and Meldolesi, 2015; Silva et al., 2017). Upon arriving at the target cells, EVs roll or “surf” on the cell surface until reaching a “hotspot” for internalization, a process driven by electrostatics and aided by membrane proteins like tetraspanins, integrins, proteoglycans, and lectins (Demonbreun and Mcnally, 2017; Meldolesi, 2018). Both, exosomes and ectosomes exhibit cell-specific signaling via different surface receptors and their ligands (Sahoo and Losordo, 2014). This is supported by the fact that presence of specific peptides in the EV membrane allows EV cargoes to be targeted to skeletal muscles or to neurons to alter the cell type (Alvarez-Erviti et al., 2011; Gao et al., 2018). Differential targeting by the EVs occurs during skeletal muscle injury and repair—EVs produced by exercise-induced muscle damage largely hone to the liver, while EVs produced from resting muscle do not do so (Whitham et al., 2018). This may be due to context-specific loading of membrane proteins within the secreted EVs that not only differ between exosomes and ectosomes, but promote different interactions with target cell membranes to ultimately cause their differential uptake (Whitham et al., 2018).

**Figure 4:**
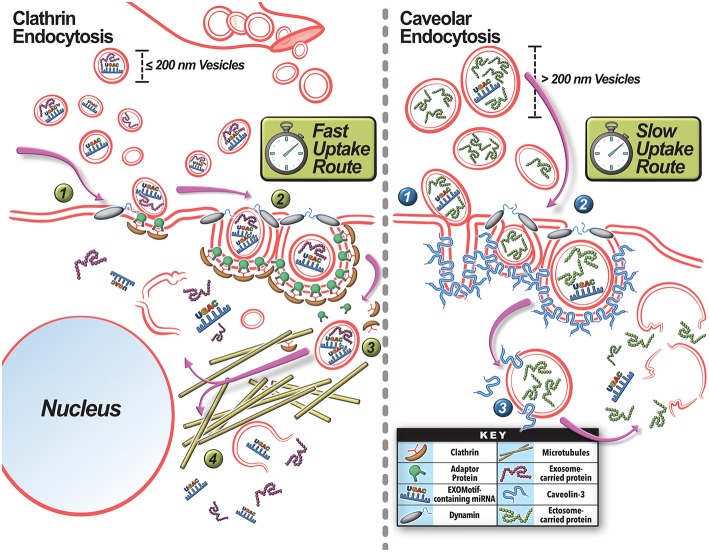
Mechanisms for extracellular vesicle uptake by the target cells. Exosomes and ectosomes produced locally or brought in via blood vessels can be taken up by **(A)** Clathrin-dependent or **(B)** Caveolin-dependent pathways. **(A)** Smaller vesicles are preferentially taken up via clathrin-mediated endocytosis. **(1)** Internalization occurs at a site for EV engagement with the plasma membrane where clathrin-coated vesicle assembly can occur by the deformation of the membrane with the help of the EV and clathrin associated adaptor proteins. **(2)** Dynamin-2 is recruited to the clathrin-pit where it forms a collar-like structure to pinch off the endosome neck. **(3)** The internalized endosome subsequently uncoats and the vesicle is shuttled via microtubules to the perinuclear region. **(4)** There the EV cargo is released. **(B)** Larger vesicles tend to favor the caveolar endocytosis pathway. Caveolar invaginations in the membrane are created by the oligomerization of Caveolin proteins. **(1)** Once the EV encounters a caveolae conducive for internalization, it engages with the caveolae. **(2)** Proteins such as Dynamin-2 and EHD2 are recruited at this site to pinch off the caveolar endosome. **(3)** Subsequently, the caveolar endosome traffics in a microtubule-independent manner and releases the EV cargo in the cytoplasm. The rate of clathrin-mediated internalization compares to rate of receptor-mediated endocytosis, and is faster than caveolae-mediated endocytosis.

Exosomes are more enriched in tetraspanins—a protein that associates with a wide range of other cell surface proteins including proteoglycans, complement-regulatory proteins, growth factor receptors and ligands (Table 1). This is important in the cellular uptake of exosomes as their membrane-embedded tetraspanins may bind more readily with a host of membrane proteins and receptors from potential target cells to facilitate their efficient uptake (Hemler, 2003; Meldolesi, 2018). In the context of muscle injury, tetraspanin-laden exosomes from injured muscle cells facilitate myoblast fusion—tetraspanin uptake by target cells induces cell spreading, and CD9 and CD81 tetraspanins specifically facilitate myoblast spreading and fusion in regeneration (Hemler, 2003). Similarly, exosomes are enriched in syncytin-1—a protein involved in receptor targeting to ILVs of the MVB that also facilitates efficient exosome uptake by target cells via binding to surface fusogens. Syncytin-1-loaded exosomes bind to their neutral amino acid transporters ASCT2 found in skeletal muscle, to induce rapid exosome internalization (Cocucci and Meldolesi, 2015; Kowal et al., 2016). In principle, these unique surface protein properties of exosomes support potentially faster uptake of exosomes (as compared to ectosomes) in muscle repair (Figure 4). Indeed, exosomes from muscle cells (myotubes and myoblasts) are taken up twice as fast by their target cells than ectosomes (Le Bihan et al., 2012). Faster uptake of exosomes also occurs in humans where damage-induced spike in exosomes was found to return to baseline within 4 h (Frühbeis et al., 2015; Callegari et al., 2017; D'souza et al., 2018). Conversely, uphill treadmill running to exhaustion under conditions that cause muscle damage (Jung et al., 2011), increases ectosome production, and they are cleared relatively slowly—their level remain elevated even after 6 h (Frühbeis et al., 2015).

The internalization pathway taken by the EV is relevant for vesicle-mediated intercellular signaling, as different endocytic pathways taken by the EV leads to different intracellular sorting and signaling fate of vesicle cargoes as well (Rejman et al., 2004; Svensson et al., 2013; Mulcahy et al., 2014; Costa Verdera et al., 2017; Schneider et al., 2017; Figure 4). This topic is discussed in detail elsewhere (Rejman et al., 2004; Le Roy and Wrana, 2005; Svensson et al., 2013; Mulcahy et al., 2014; Tian et al., 2014; Costa Verdera et al., 2017; Schneider et al., 2017). Interestingly, altering the intensity and damaging-potential of exercise can shift EV production to favor exosome secretion (Oliveira et al., 2018). This may be a mechanism to confer a more rapid repair and regenerative response from surrounding target cells after a more damaging stimulus—as exosomes may be more efficiently taken up (Dittrich et al., 2013). Different pathways for exosome and ectosome uptake also impact on their intracellular shuttling and thus potential intracellular site of action by their cargoes. Clathrin-mediated uptake of smaller vesicles uses microtubules (Svensson et al., 2013) to shuttle these cargoes to the perinuclear region of the cell, while the caveolar endocytosis of larger vesicles shuttles them to the cell periphery in a microtubule-independent manner (Figure 4; Rejman et al., 2004). Thus, the differences in size and membrane composition of EVs could influence how, when, and where these EVs are taken up and delivered to in the cell. This could then alter the nature of intercellular signaling each of them can engage in during muscle repair and regeneration. Thus, in addition to selective cargo packaging, use of specific EVs allows the injured myofibers control over modulating the timing, site of action, and intracellular location of the signals they communicate to other cells in the injured tissue.

## Extracellular Vesicle-Mediated Communication During Muscle Repair and Regeneration

Co-ordinated extracellular interactions between inflammatory cells, endothelial cells, mesenchymal stem cells (MSCs), and myogenic stem cells are critical for regeneration of injured skeletal myofibers (Wosczyna and Rando, 2018). EV-mediated transfer of cargoes facilitates repair and regeneration across tissues including cardiac, intestinal, neural, renal, respiratory, and skeletal muscles (Cocucci et al., 2009; Leoni et al., 2013, 2015; Lopez-Verrilli et al., 2013; Demonbreun and Mcnally, 2017; Taverna et al., 2017). These diverse EV cargoes help alter the phenotype of the recipient cells by affecting the recipient cell's mRNA composition, translation, or by directly transferring membrane components such as receptors, transmembrane proteins, and lipids to activate downstream pathways involved in maintenance of tissue homeostasis and repair (Cocucci et al., 2009; Taverna et al., 2017). Below, we discuss how immediately following muscle injury EVs facilitate “myofiber repair” and later on facilitate “myofiber regeneration” to restore the lost muscle tissue.

### Extracellular Vesicles in Myofiber Repair and Early Stages of Muscle Regeneration

While much of the work on EVs has focused on intercellular communication during tissue regeneration, recent studies have started to address their role in facilitating repair of injured cells. As discussed above, cell membrane damage leads to acute actin reorganization and membrane remodeling through the action of mitochondrial redox signaling, annexin binding, and ESCRT activity (Jaiswal et al., 2004, 2014; Bouter et al., 2011; Scheffer et al., 2014; Boye et al., 2017; Horn et al., 2017). Together, these help close the membrane wounds within minutes of being injured. These early stages of repair can be directly influenced by EVs. For example, intestinal epithelial cell injury causes secretion of Annexin-A1 containing vesicles that can induce redox signaling via Nox1, which act on Rac, and p120 proteins to enable faster closure of the epithelial wound (Leoni et al., 2013, 2015). The N-terminal Annexin A1 peptide Ac2-26 enables epithelial wound repair by binding formyl peptide receptor (FPR1) that activate downstream focal adhesion protein Paxillin and focal adhesion kinase (FAK) to stimulate cell migration, and wound closure (Bizzarro et al., 2012a; Leoni et al., 2015). Skeletal muscles also express these FPRs, which are crucial during regenerative stages in stimulating myoblast proliferation by exosome-derived Annexin-A1 (Bizzarro et al., 2012b). Additionally, myofiber sarcolemmal injury causes several Annexins—A1, A2, A5, and A6, to accumulate at or outside of the injured sarcolemma and some of these are also shown to be required for myofiber repair (Roostalu and Strähle, 2012; Demonbreun et al., 2016; D'souza et al., 2018; Hogarth et al., 2019). Annexin A1 is the first to accumulate at the injury site and is shed by the damaged plasma membrane by action of Annexin A2-mediated actin polymerization (Jaiswal et al., 2014). Mice lacking Annexin A2 and those expressing truncated dominant negative form of Annexin A6 poorly repair myofiber injury, implicating Annexins in vesicle shedding and other intracellular vesicle trafficking processes important for repair (Swaggart et al., 2014; D'souza et al., 2018). Interestingly, mice lacking Annexin A1 do not show defects in membrane repair, but are affected in their ability to efficiently regenerate damaged myofibers (Leikina et al., 2015). Thus, while annexins are amongst the earliest responders to myofiber injury, their role extends beyond myofiber repair to myofiber regeneration. Such a role of annexins is supported by the recent finding that annexin A2, an ectosome-enriched protein, which is required for myofiber repair, also influences the muscle resident immune and other cells that leads to adipogenic loss of muscles in Limb Girdle Muscular Dystrophy (LGMD) 2B—a disease associated with poor myofiber repair due to the lack of dysferlin protein (D'souza et al., 2018; Hogarth et al., 2019).

Shedding of vesicles by injured myofibers, muscle and other cells, is enabled by ESCRT-mediated ectosome formation at the plasma membrane. Failure of this process prevents repair of the injured plasma membrane (Jimenez et al., 2014; Scheffer et al., 2014). Unlike ILV formation at the MVB, where the ESCRTs I and II are required for the assembly of ESCRT III to mediate ILV budding and scission, ectosome budding, and scission at the site of injury is initiated by the calcium binding protein ALG-2 (Apoptosis linked gene-2) (Scheffer et al., 2014). ALG-2 accumulates at this injury site in response to the local influx of calcium and it in turn recruits its binding partner—ALG-2 interacting protein X (ALIX), to localize ESCRT III and Vps4 ATPase that cleaves the nascent ectosome (Scheffer et al., 2014). Formation of outwardly budding vesicle at the site of sarcolemmal injury has been observed in intact muscles and also in isolated skeletal muscle fibers, where the vesicle formation involves a coordinated action of multiple proteins including annexins as well as membrane proteins and actin cytoskeleton (Scheffer et al., 2014; Demonbreun et al., 2016; Figure 1; Video S1). Shedding of damaged membrane at the site of sarcolemmal injury is also observed in Zebrafish, where the membrane repair protein dysferlin is reported to accumulate sarcolemmal phosphatidylserine to the repair site to be recognized and removed through phagocytosis (Middel et al., 2016). Failure of sarcolemmal vesiculation due to the lack of ESCRTs or actin, dysferlin, and other accessory proteins that help with the proper membrane dynamics and outward budding of the injured sarcolemma prevents repair of the injured myofibers (Scheffer et al., 2014).

In addition to facilitating myofiber repair the extracellular vesicles shed by the repairing myofiber and other cells also initiate extracellular interactions for muscle tissue regeneration (Figure 2). For instance, EVs trigger pro-inflammatory cascades through transport of antigens loaded onto MHC class 1 and 2 complexes to T cells, as part of the initial inflammatory events required for regeneration following skeletal muscle injury (Taverna et al., 2017). Similarly, hypoxia-injured myotubes produce vesicles that increase macrophage expression of inflammatory IL-6 that in-turn alters myogenesis (Guescini et al., 2017). Initial invading neutrophils with skeletal muscle injury, migrate to the site of damage and release ectosome vesicles that contain F-actin, and membrane-bound Annexin-5, selectins, integrins, complement regulator HLA-1, and matrix metalloproteinases (MMPs) that degrade the ECM, and preferentially bind to monocytes to propagate the inflammatory response (Gasser et al., 2003; Taverna et al., 2017). Additionally, neutrophil-derived ectosomes stimulate the release of anti-inflammatory factors (TGF-β1, and IL-10) from macrophages to fine-tune inflammatory pre-conditioning for eventual pro-inflammatory macrophage induction (Gasser et al., 2003; Tidball, 2017). At later timepoints after injury, ectosomes transfer chemokine receptors (CCR4, CCR5), and stimulate release of IL-6 and monocyte chemotactic protein 1 (MCP1) that promote inflammation (Gasser et al., 2003). However, macrophages are not merely recipients of these EVs, as seen during vascular endothelial cell injury where macrophages secrete ectosome vesicles that bind platelets to initiate the coagulation cascade and wound healing (Del Conde et al., 2005). With muscle membrane injury, the acute mitochondrial ROS production near the site of injury coincides with actin buildup and ectosome release that peaks within 2–4 min post-injury (Scheffer et al., 2014; Horn et al., 2017; Figures 1C,D). With their production near the injury site where mitochondrial ROS levels are the highest, ectosomes are likely to contain oxidized plasma membrane lipids which can stimulate neighboring TLR-expressing macrophages to promote inflammation and cell repair (Zhou et al., 2018). Interestingly, miRNAs delivered via exosomes, reduce macrophage expression of TLRs, and cause these cells to take-up other vesicles and their cargoes without immune activation, a vesicle-signaling effect demonstrated in muscular dystrophy (Mancek-Keber et al., 2015; Phinney et al., 2015; Hindi and Kumar, 2016). Such findings raise the intriguing possibility that exosomes and ectosomes may act in coordinated ways to fine-tune their intercellular signals and cellular stimulation in muscle repair and regeneration.

### Extracellular Vesicles in Myofiber Regeneration and Restoration of Muscle Damage

As discussed in section Events that Facilitate Myofiber Repair and Regeneration, injuries that cause myofiber death require regeneration of these myofibers. This occurs through a program that first clears the cellular debris and then guides the regenerative response (Chazaud, 2016). This program involves release of neutrophil-attractants CXCL1 and CCL2, which enrich the muscle environment in cytokines that activate macrophages to a pro-inflammatory phenotype (Tidball, 2017). These pro-inflammatory cytokines cause myotubes to increase production of exosomes that are loaded with the myostatin protein, while concomitantly decreasing the level of packaged myostatin antagonist protein decorin (Kim et al., 2018). These exosomes inhibit myogenic regulatory factors (MRFs)—myoD and myogenin expression and Akt and mTOR-mediated myogenesis, while increasing myoblast COX-2 expression (Kim et al., 2018). Thus, at this early pro-inflammatory stage of muscle repair, exosomes participate in limiting myogenesis and allowing the inflammatory cells to clear the damaged tissue. This is followed by a change in macrophage polarization to pro-regenerative state with a concomitant rise in MSCs (Tidball, 2017; Wosczyna and Rando, 2018). These MSCs secrete vesicles that stimulate MYOD, and Myogenin, which facilitate myofiber regeneration in target cells (Phinney et al., 2015). Moreover, MSC exosomes attenuate fibrosis, improve capillary density, and accelerate regeneration of the injured muscle (Nakamura et al., 2015). This is largely accomplished via cellular transfer of vesicle cargo VEGF, and IL-6, in addition to exosome-enriched miR-1, miR-133, miR-206 (containing EXOmotif's GGAC, CCCU, CCCG, respectively), and miR-125b, miR-494, and miR-601, that promote a variety of pro-regenerative cellular processes (Table 3). However, these MSCs not only transfer pro-regenerative vesicles to injured skeletal muscle cells and SCs, they can also package and transfer mitochondria and mitochondrial proteins within ectosomes, to the invading macrophages. This is suggested to enhance macrophage energetics and inflammatory activity vital for regeneration (Phinney et al., 2015; Sansone et al., 2017).

Exosomes also facilitate myogenesis in other muscle injury contexts. Skeletal muscle denervation injury shifts the muscle-derived EV enrichment from miR-133a and miR-720 to the EXOmotif containing miRNA—miR-206. miR-206 stimulates satellite cell differentiation, myofiber maturation, and brain-derived neurotrophic factor (BDNF), and nerve-growth factor (NGF) production, which together enhance myofiber re-innervation (De Gasperi et al., 2017). These pro-myogenic and neuro-regenerative effects of miRNA-206 offer further evidence for the benefits of context-specific EV cargo loading to facilitate distinct regenerative effects (Mccarthy, 2008; Yuasa et al., 2008). SC differentiation during regeneration, also actively increases their secretion of vesicles that contain growth factors (IGF-1, HGF, TGF-B1, FGF2, VEGF, and PDGF) that act in SC chemotaxis, lineage commitment, and neovascularization, while simultaneously transferring miRNA cargoes such as miR-206, and miR-1 that promote SC differentiation by altering myogenic gene expression in neighboring SCs (Braun and Gautel, 2011; Forterre et al., 2014; Murphy et al., 2018). Additionally, exosomes are also released by differentiating satellite cells to help attenuate fibrosis and enhance myofiber regeneration via cargo transfer (Braun and Gautel, 2011; Forterre et al., 2014; Choi et al., 2016; Murphy et al., 2018). Indeed, skeletal muscles injured by laceration, secrete exosomes enriched in myogenic growth factors that subsequently stimulate differentiation of adipose-derived stem cells toward a myogenic lineage to assist in muscle regeneration (Choi et al., 2016). Similarly, oxidatively injured myotubes promote satellite cell proliferation by vesicle-mediated repression of myogenin expression in target satellite cells, resulting in faster wound closure in an *in-vitro* wound assay (Guescini et al., 2017).

Similar to the integral role of vesicles in the regenerative mobilization of tissue resident stem cells, they are also involved in remodeling of damaged tissue by facilitating angiogenesis, fibroblast activation, deposition, and degradation of new ECM, and tissue cell replenishment. Skeletal muscle remodeling involves dynamic changes in extracellular matrix structure and composition. Indeed, exosomes isolated from SCs after overload-induced muscle damage downregulate collagen and fibronectin production by neighboring fibroblasts through the transfer of their miR-206 cargo that inhibits fibroblast Rrbp1—the master regulator of collagen and fibrogenic expression (Fry et al., 2017). This attenuated ECM deposition can then help damaged muscle regenerate and remodel for hypertrophic adaptation. Similarly, high intensity cycling-induced muscle damage induces enrichment in 12 miRNAs within the exosome pool which did not concomitantly increase in the plasma or within the cells themselves, nor was there a global elevation in exosomal miRNAs—suggesting that these miRNAs were neither passively leaked, nor part of a general increase in exosomal miRNA abundance (D'souza et al., 2018). Many of these 12 miRNAs are known to fine-tune muscle regeneration. During muscle regeneration and remodeling, miR-208a regulates fiber type determination, while miR-126 and miR-16 regulate blood vessel formation (D'souza et al., 2018). These findings support the aforementioned evidence that specific miRNAs may be transcribed by the cell under certain cellular stresses to accomplish highly specific and targeted intercellular signaling goals in myofiber regeneration and remodeling. Both miR-126 and miR-16 contain the UGAC and GGCG EXOmotifs, respectively, and stimulate neovascularization in response to an aerobically taxing and damaging stimulus- implicating these motifs in context-specific and selective packaging in exosomes based on the nature of the stimulus/damage. This specific miRNA production and packaging in exosomes shows that under certain cellular stresses, EVs accomplish specific intercellular signaling goals to enable myofiber regeneration and remodeling. This is not limited to skeletal muscles alone however, as in the early stages of cardiac muscle hypertrophy, the mechanical stress and damage to cardiomyocytes promote secretion of miRNA-378-containing EVs that impair fibroblast hyperplasia and attenuate their production of collagen, to limit cardiac fibrosis (Yuan et al., 2018). These findings implicate specific miRNA transcription by the cell under certain cellular stresses to accomplish highly specific intercellular signaling goals in muscle regeneration and remodeling.

In addition to cargo packaging, purposeful and context-specific release of EVs also regulates contextual signaling by EVs. Unlike damage to healthy muscles, damage associated with Duchenne muscular dystrophy causes muscle-resident fibroblasts to secrete vesicles with increased levels of miR-199a-5p that promotes increased fibrosis in skeletal muscle and surrounding matrix (Zanotti et al., 2018). Similarly, vesicles from the serum of *mdx* mice, when administered to myoblasts, promote survival, and reduce cell death (Murphy et al., 2018). These *mdx* exosomes protect against cell death in muscle cells stressed by excessive reactive oxygen species—in a manner that is linearly associated with the concentration of these vesicles (Matsuzaka et al., 2016). This pro-survival effect of EVs was achieved by the exosome transfer of miR-133a—a miRNA that decreases caspase-mediated proteolysis and decreases expression of apoptosis-associated genes (Matsuzaka et al., 2016). Conversely, inhibiting exosome formation in the MVB (by neutral sphingomyelinase inhibition), or blocking vesicle uptake by target cells via reduction of membrane cholesterol with MBCD treatment, each abolished the protective effect of *mdx* muscle exosomes in hypoxia-stressed muscle cells (Matsuzaka et al., 2016). Interestingly, pro-myogenic miRNA cargoes (miRNAs−1, 133a, and 206) in exosomes produced by *mdx* muscles, are absent from exosomes from muscles where dystrophin has been restored (Roberts et al., 2012). This suggests that muscle instability-induced damage results in selective production and packaging of exosomes to promote regenerative effects, which can be attenuated by improved muscle stability. This exemplifies cell-stress specific vesicle cargo production and packaging. Supporting this feature of *mdx* EVs, dystrophin-deficient cardiomyocytes also produce not only smaller EVs than healthy cardiomyocytes, but these vesicles have the unique capacity to protect neighboring cardiomyocytes from cell death following their uptake (Gartz et al., 2018).

## Conclusion

It has become increasingly clear from the mounting body of evidence in skeletal muscle injury, that EV secretion and extracellular signaling occurs along the continuum of muscle repair and regeneration. The studies discussed above suggest the existence of an injury-responsive production of EVs packaged with proteins, RNAs, and lipids to facilitate repair and regeneration. This cargo packaging appears to be selective based upon the context and type of injury, required intercellular signaling, and intended cellular targets, thus adding a previously unrecognized layer of complexity to this process beyond the initially postulated cell debris hypothesis. While exosomes and ectosomes are similar in their basic structure and range of cargoes, these vesicles differ in nearly all critical elements—specific cargo, route of cellular uptake, rate of uptake, intracellular handling by the target cell, and time course of secretion by the parent cell. While EVs are produced actively, and their signaling effects help orchestrate the continuum of response from myofiber repair to regeneration, this complex diversity of EVs in the context of healthy and diseased muscle repair needs to be acknowledged in muscle physiology research. These features of EVs raise additional mechanistic questions—how does alteration in total ectosome and exosome production, rate of secretion, and cargoes improve or impair health of injured muscles? Does the improved repair capacity of skeletal muscle with repeated insult (i.e., repeated bout effect of eccentric exercise) derive, in-part, from alterations in vesicle production, secretion, and or cargo loading? Answers to such questions may build upon the current knowledge discussed here, and may highlight the roles of EVs being provided by other tissues and physiological processes. Such findings are bound to offer exciting new insights into the coordination of the complex process of muscle repair, and how this can be optimized for therapeutic purposes in disease as well as in sport.

## Author Contributions

DB and JJ contributed to the conception and writing of this paper.

### Conflict of Interest Statement

The authors declare that the research was conducted in the absence of any commercial or financial relationships that could be construed as a potential conflict of interest.

## Supplementary Material

Video S1 Real-time visualization of the release of small and large vesicles by injured skeletal myofibers. Confocal time lapse video shows a pair of live myofibers in an intact mouse biceps muscle injured focally via pulsed laser. Myofiber sarcolemma and released vesicles are labeled with green membrane binding dye FM 1-43 present in the buffer. Laser injury occurs at 20 s into the video—note the secretion of vesicles from the site of injury, which then travel/accumulate in the inter-fiber space. The time is shown in min:ss.msec format and the scale bar represent 10 μm.

## References

[B1] Aicart-Ramos C. Valero R. A. Rodriguez-Crespo I. (2011). Protein palmitoylation and subcellular trafficking. Biochim. Biophys. Acta 1808, 2981–2994. 10.1016/j.bbamem.2011.07.009 21819967

[B2] Alvarez-Erviti L. Seow Y. Yin H. Betts C. Lakhal S. Wood M. J. (2011). Delivery of siRNA to the mouse brain by systemic injection of targeted exosomes. Nat. Biotechnol. 29, 341–345. 10.1038/nbt.1807 21423189

[B3] Andrews N. W. Almeida P. E. Corrotte M. (2014). Damage control: cellular mechanisms of plasma membrane repair. Trends Cell Biol. 24, 734–742. 10.1016/j.tcb.2014.07.008 25150593 PMC4252702

[B4] Appleyard S. T. Dunn M. J. Dubowitz V. Rose M. L. (1985). Increased expression of HLA ABC class I antigens by muscle fibres in duchenne muscular dystrophy, inflammatory myopathy, and other neuromuscular disorders. Lancet 1, 361–363. 10.1016/S0140-6736(85)91384-4 2857418

[B5] Arkhipov A. Yin Y. Schulten K. (2008). Four-scale description of membrane sculpting by BAR domains. Biophys. J. 95, 2806–2821. 10.1529/biophysj.108.132563 18515394 PMC2527247

[B6] Azakir B. A. Di Fulvio S. Therrien C. Sinnreich M. (2010). Dysferlin interacts with tubulin and microtubules in mouse skeletal muscle. PLoS ONE 5:e10122. 10.1371/journal.pone.0010122 20405035 PMC2853571

[B7] Babiychuk E. B. Draeger A. (2000). Annexins in cell membrane dynamics. Ca(2+)-regulated association of lipid microdomains. J. Cell Biol. 150, 1113–1124. 10.1083/jcb.150.5.1113 10973999 PMC2175252

[B8] Babiychuk E. B. Monastyrskaya K. Potez S. Draeger A. (2011). Blebbing confers resistance against cell lysis. Cell Death Differ. 18, 80–89. 10.1038/cdd.2010.81 20596076 PMC3131879

[B9] Bach A. S. Enjalbert S. Comunale F. Bodin S. Vitale N. Charrasse S.. (2010). ADP-ribosylation factor 6 regulates mammalian myoblast fusion through phospholipase d1 and phosphoatidylinositol 4,5-bisphosphate signaling pathways. Mol Biol Cell 21, 2412–2424. 10.1091/mbc.e09-12-1063 20505075 PMC2903670

[B10] Badawy S. M. M. Okada T. Kajimoto T. Hirase M. Matovelo S. A. Nakamura S.. (2018). Extracellular α-synuclein drives sphingosine 1-phosphate receptor subtype 1 out of lipid rafts, leading to impaired inhibitory G-protein signaling. J. Biol. Chem. 293, 8208–8216. 10.1074/jbc.RA118.001986 29632069 PMC5971450

[B11] Benink H. A. Bement W. M. (2005). Concentric zones of active RhoA and Cdc42 around single cell wounds. J. Cell Biol. 168, 429–439. 10.1083/jcb.200411109 15684032 PMC2171735

[B12] Bennet A. H. O'Donogue M. F. Gundry S. R. Chan A. T. Widrick J. Draper I. (2018). RNA helicase, DDX27 regulates skeletal muscle growth and regeneration by modulation of translational processes. PLoS Genet. 14:e1007226 10.1371/journal.pgen.1007226 29518074 PMC5843160

[B13] Bentzinger C. F. Wang Y. X. Dumont N. A. Rudnicki M. A. (2013). Cellular dynamics in the muscle satellite cell niche. EMBO Rep. 14, 1062–1072. 10.1038/embor.2013.182 24232182 PMC3849491

[B14] Berger J. Berger S. Li M. Jacoby A. S. Arner A. Bavi N. (2018). *In vivo* function of the chaperonin tric in a-actin folding during sarcomere assembly. Cell Rep. 22, 313–322. 10.1016/j.celrep.2017.12.069 29320728

[B15] Bianco F. Perrotta C. Novellino L. Francolini M. Riganti L. Menna E.. (2009). Acid sphingomyelinase activity triggers microparticle release from glial cells. EMBO J. 28, 1043–1054. 10.1038/emboj.2009.45 19300439 PMC2664656

[B16] Bizzarro V. Belvedere R. Dal Piaz F. Parente L. Petrella A. (2012a). Annexin A1 induces skeletal muscle cell migration acting through formyl peptide receptors. PLoS ONE 7:e48246. 10.1371/journal.pone.0048246 23144744 PMC3483218

[B17] Bizzarro V. Petrella A. Parente L. (2012b). Annexin A1: novel roles in skeletal muscle biology. J. Cell Physiol. 227, 3007–3015. 10.1002/jcp.24032 22213240

[B18] Blanc M. David F. Abrami L. Migliozzi D. Armand F. Bürgi J.. (2015). SwissPalm: protein palmitoylation database. F1000Res. 4:261. 10.12688/f1000research.6464.1 26339475 PMC4544385

[B19] Bouter A. Gounou C. Bérat R. Tan S. Gallois B. Granier T.. (2011). Annexin-A5 assembled into two-dimensional arrays promotes cell membrane repair. Nat. Commun. 2:270. 10.1038/ncomms1270 21468022 PMC3104517

[B20] Boye T. L. Jeppesen J. C. Maeda K. Pezeshkian W. Solovyeva V. Nylandsted J.. (2018). Annexins induce curvature on free-edge membranes displaying distinct morphologies. Sci. Rep. 8:10309. 10.1038/s41598-018-28481-z 29985397 PMC6037701

[B21] Boye T. L. Maeda K. Pezeshkian W. Sønder S. L. Haeger S. C. Gerke V.. (2017). Annexin A4 and A6 induce membrane curvature and constriction during cell membrane repair. Nat. Commun. 8:1623. 10.1038/s41467-017-01743-6 29158488 PMC5696365

[B22] Braun T. Gautel M. (2011). Transcriptional mechanisms regulating skeletal muscle differentiation, growth and homeostasis. Nat. Rev. Mol. Cell Biol. 12, 349–361. 10.1038/nrm3118 21602905

[B23] Cai C. Masumiya H. Weisleder N. Matsuda N. Nishi M. Hwang M. (2009). MG53 nucleates assembly of cell membrane repair machinery. Nat. Cell Biol. 11, 56–64. 10.1038/ncb1812 19043407 PMC2990407

[B24] Callegari G. A. Novaes J. S. Neto G. R. Dias I. Garrido N. D. Dani C. (2017). Creatine kinase and lactate dehydrogenase responses after different resistance and aerobic exercise protocols. J. Hum. Kinet. 58, 65–72. 10.1515/hukin-2017-0071 28828078 PMC5548155

[B25] Carson J. A. Wei L. (2000). Integrin signaling's potential for mediating gene expression in hypertrophying skeletal muscle. J. Appl. Physiol. 88, 337–343. 10.1152/jappl.2000.88.1.337 10642399

[B26] Caruso S. Poon I. K. H. (2018). Apoptotic cell-derived extracellular vesicles: more than just debris. Front. Immunol. 9:1486. 10.3389/fimmu.2018.01486 30002658 PMC6031707

[B27] Chakrabarti S. Kobayashi K. S. Flavell R. A. Marks C. B. Miyake K. Liston D. R.. (2003). Impaired membrane resealing and autoimmune myositis in synaptotagmin VII-deficient mice. J. Cell Biol. 162, 543–549. 10.1083/jcb.200305131 12925704 PMC2173791

[B28] Chazaud B. (2016). Inflammation during skeletal muscle regeneration and tissue remodeling: application to exercise-induced muscle damage management. Immunol. Cell Biol. 94, 140–145. 10.1038/icb.2015.97 26526620

[B29] Chen J. F. Tao Y. Li J. Deng Z. Yan Z. Xiao X.. (2010). microrna-1 and microrna-206 regulate skeletal muscle satelite cell proliferation and differentiation by repressing pax7. J. Cell Biol. 190, 867–879. 10.1083/jcb.200911036 20819939 PMC2935565

[B30] Chen M. Xu R. Ji H. Greening D. W. Rai A. Izumikawa K.. (2016). Transcriptome and long noncoding RNA sequencing of three extracellular vesicle subtypes released from the human colon cancer LIM1863 cell line. Sci. Rep. 6:38397. 10.1038/srep38397 27917920 PMC5137021

[B31] Choi J. S. Yoon H. I. Lee K. S. Choi Y. C. Yang S. H. Kim I. S.. (2016). Exosomes from differentiating human skeletal muscle cells trigger myogenesis of stem cells and provide biochemical cues for skeletal muscle regeneration. J. Control Release 222, 107–115. 10.1016/j.jconrel.2015.12.018 26699421

[B32] Cocucci E. Meldolesi J. (2015). Ectosomes and exosomes: shedding the confusion between extracellular vesicles. Trends Cell Biol. 25, 364–372. 10.1016/j.tcb.2015.01.004 25683921

[B33] Cocucci E. Racchetti G. Meldolesi J. (2009). Shedding microvesicles: artefacts no more. Trends Cell Biol. 19, 43–51. 10.1016/j.tcb.2008.11.003 19144520

[B34] Coenen-Stass A. M. Betts C. A. Lee Y. F. Mäger I. Turunen M. P. El Andaloussi S.. (2016). Selective release of muscle-specific, extracellular microRNAs during myogenic differentiation. Hum. Mol. Genet. 25, 3960–3974. 10.1093/hmg/ddw237 27466195 PMC5291232

[B35] Colombo M. Raposo G. Théry C. (2014). Biogenesis, secretion, and intercellular interactions of exosomes and other extracellular vesicles. Annu. Rev. Cell Dev. Biol. 30, 255–289. 10.1146/annurev-cellbio-101512-122326 25288114

[B36] Conti F. J. Monkley S. J. Wood M. R. Critchley D. R. Muller U. (2009). Talin 1 and 2 are required for myoblast fusion, sarcomere assembly and the maintenance of myotendinous junctions. Development 136, 3597–3606. 10.1242/dev.035857 19793892 PMC2761109

[B37] Cooper S. T. Mcneil P. L. (2015). Membrane repair: mechanisms and pathophysiology. Physiol. Rev. 95, 1205–1240. 10.1152/physrev.00037.2014 26336031 PMC4600952

[B38] Corrotte M. Almeida P. E. Tam C. Castro-Gomes T. Fernandes M. C. Millis B. A.. (2013). Caveolae internalization repairs wounded cells and muscle fibers. Elife 2:e00926. 10.7554/eLife.00926 24052812 PMC3776555

[B39] Costa Verdera H. Gitz-Francois J. J. Schiffelers R. M. Vader P. (2017). Cellular uptake of extracellular vesicles is mediated by clathrin-independent endocytosis and macropinocytosis. J. Control Release 266, 100–108. 10.1016/j.jconrel.2017.09.019 28919558

[B40] Counsel P. Breidahl W. (2010). Muscle injuries of the lower leg. Semin. Musculoskelet. Radiol. 14, 162–175. 10.1055/s-0030-1253158 20486025

[B41] Crescitelli R. Lässer C. Szabó T. G. Kittel A. Eldh M. Dianzani I.. (2013). Distinct RNA profiles in subpopulations of extracellular vesicles: apoptotic bodies, microvesicles and exosomes. J. Extracell. Vesicles 2:20677. 10.3402/jev.v2i0.20677 24223256 PMC3823106

[B42] De Gasperi R. Hamidi S. Harlow L. M. Ksiezak-Reding H. Bauman W. A. Cardozo C. P. (2017). Denervation-related alterations and biological activity of miRNAs contained in exosomes released by skeletal muscle fibers. Sci. Rep. 7:12888. 10.1038/s41598-017-13105-9 29038428 PMC5643439

[B43] Defour A. Van Der Meulen J. H. Bhat R. Bigot A. Bashir R. Nagaraju K.. (2014). Dysferlin regulates cell membrane repair by facilitating injury-triggered acid sphingomyelinase secretion. Cell Death Dis. 5:e1306. 10.1038/cddis.2014.272 24967968 PMC4079937

[B44] Del Conde I. Shrimpton C. N. Thiagarajan P. López J. A. (2005). Tissue-factor-bearing microvesicles arise from lipid rafts and fuse with activated platelets to initiate coagulation. Blood 106, 1604–1611. 10.1182/blood-2004-03-1095 15741221

[B45] Demonbreun A. R. Mcnally E. M. (2017). Muscle cell communication in development and repair. Curr. Opin. Pharmacol. 34, 7–14. 10.1016/j.coph.2017.03.008 28419894 PMC5641474

[B46] Demonbreun A. R. Quattrocelli M. Barefield D. Y. Allen M. V. Swanson K. E. Mcnally E. M. (2016). An actin-dependent annexin complex mediates plasma membrane repair in muscle. J. Cell. Biol. 213, 705–718. 10.1083/jcb.201512022 27298325 PMC4915191

[B47] Deng L. Peng Y. Jiang Y. Wu Y. Ding Y. Wang Y.. (2017). Imipramine protects against bone loss by inhibition of osteoblast-derived microvesicles. Int. J. Mol. Sci. 18:1013. 10.3390/ijms18051013 28481322 PMC5454926

[B48] Di Filippo E. S. Mancinelli R. Pietrangelo T. La Rovere R. M. Quattrocelli M. Sampaolesi M.. (2016). Myomir dysregulation and reactive oxygen species in aged human satellite cells. Biochem. Biophys. Res. Commun. 473, 462–470. 10.1016/j.bbrc.2016.03.030 26975470

[B49] Dittrich N. De Lucas R. D. Maioral M. F. Diefenthaeler F. Guglielmo L. G. (2013). Continuous and intermittent running to exhaustion at maximal lactate steady state: neuromuscular, biochemical and endocrinal responses. J. Sci. Med. Sport. 16, 545–549. 10.1016/j.jsams.2012.12.001 23391432

[B50] Dobro M. J. Samson R. Y. Yu Z. Mccullough J. Ding H. J. Chong P. L.. (2013). Electron cryotomography of ESCRT assemblies and dividing Sulfolobus cells suggests that spiraling filaments are involved in membrane scission. Mol. Biol. Cell 24, 2319–2327. 10.1091/mbc.e12-11-0785 23761076 PMC3727925

[B51] Donati C. Cencetti F. Bruni P. (2013). Sphingosine 1-phosphate axis: a new leader actor in skeletal muscle biology. Front. Physiol. 4:338. 10.3389/fphys.2013.00338 24324439 PMC3839259

[B52] Draeger A. Babiychuk E. B. (2013). Ceramide in plasma membrane repair. Handb Exp. Pharmacol. 216, 341–353. 10.1007/978-3-7091-1511-4_17 23563665

[B53] D'souza A. Medikayala S. Van Der Meulen J. H. Hogarth M. W. Holdreith N. Malatras A. (2018). Annexin A2 links poor myofiber repair with inflammation and adipogenic replacement of the injured muscle. Hum. Molecul. Genet. 26, 1979–1991. 10.1093/hmg/ddx065 PMC6075559 28334824

[B54] D'souza R. F. Woodhead J. S. T. Zeng N. Blenkiron C. Merry T. L. Cameron-Smith D.. (2018). Circulatory exosomal miRNA following intense exercise is unrelated to muscle and plasma miRNA abundances. Am. J. Physiol. Endocrinol. Metab. 315, E723–E733. 10.1152/ajpendo.00138.2018 29969318

[B55] Duan X. Chan K. T. Lee K. K. Mak A. F. (2015). Oxidative stress and plasma membrane repair in single myoblasts after femtosecond laser photoporation. Ann. Biomed. Eng. 43, 2735–2744. 10.1007/s10439-015-1341-4 26014361

[B56] Edgar J. R. (2016). Q&A: what are exosomes, exactly? BMC Biol. 14:46. 10.1186/s12915-016-0268-z 27296830 PMC4906597

[B57] Eliseeva I. A. Kim E. R. Guryanov S. G. Ovchinnikov L. P. Lyabin D. N. (2011). Y-box-binding protein (YB-1) and its functions. Biochemistry (Mosc.) 76, 1402–1433. 10.1134/S0006297911130049 22339596

[B58] Floyd C. L. Rzigalinski B. A. Weber J. T. Sitterding H. A. Willoughby K. A. Ellis E. F. (2001). Traumatic injury of cultured astrocytes alters inositol (1,4,5)-trisphosphate-mediated signaling. Glia 33, 12–23. 10.1002/1098-1136(20010101)33:1%3C12::AID-GLIA1002%3E3.0.CO;2-V 11169788

[B59] Forterre A. Jalabert A. Chikh K. Pesenti S. Euthine V. Granjon A.. (2014). Myotube-derived exosomal miRNAs downregulate Sirtuin1 in myoblasts during muscle cell differentiation. Cell Cycle 13, 78–89. 10.4161/cc.26808 24196440 PMC3925739

[B60] Frenette J. Chbinou N. Godbout C. Marsolais D. Frenette P. S. (2003). Macrophages, not neutrophils, infiltrate skeletal muscle in mice deficient in P/E selectins after mechanical reloading. Am. J. Physiol. Regul. Integr. Comp. Physiol. 285, R727–R732. 10.1152/ajpregu.00175.2003 12829442

[B61] Frühbeis C. Helmig S. Tug S. Simon P. Krämer-Albers E. M. (2015). Physical exercise induces rapid release of small extracellular vesicles into the circulation. J. Extracell Vesicles 4:28239. 10.3402/jev.v4.28239 26142461 PMC4491306

[B62] Fry C. S. Kirby T. J. Kosmac K. Mccarthy J. J. Peterson C. A. (2017). Myogenic progenitor cells control extracellular matrix production by fibroblasts during skeletal muscle hypertrophy. Cell Stem Cell 20, 56–69. 10.1016/j.stem.2016.09.010 27840022 PMC5218963

[B63] Gao X. Ran N. Dong X. Zuo B. Yang R. Zhou Q.. (2018). Anchor peptide captures, targets, and loads exosomes of diverse origins for diagnostics and therapy. Sci. Transl. Med. 10:eaat0195. 10.1126/scitranslmed.aat0195 29875202

[B64] Gartz M. Darlington A. Afzal M. Z. Strande J. L. (2018). Exosomes exert cardioprotection in dystrophin-deficient cardiomyocytes via ERK1/2-p38/MAPK signaling. Sci. Rep. 8:16519. 10.1038/s41598-018-34879-6 30410044 PMC6224575

[B65] Gasser O. Hess C. Miot S. Deon C. Sanchez J. C. Schifferli J. A. (2003). Characterisation and properties of ectosomes released by human polymorphonuclear neutrophils. Exp. Cell Res. 285, 243–257. 10.1016/S0014-4827(03)00055-7 12706119

[B66] Gitler D. Spira M. E. (1998). Real time imaging of calcium-induced localized proteolytic activity after axotomy and its relation to growth cone formation. Neuron 20, 1123–1135. 10.1016/S0896-6273(00)80494-8 9655501

[B67] Guescini M. Maggio S. Ceccaroli P. Battistelli M. Annibalini G. Piccoli G.. (2017). Extracellular vesicles released by oxidatively injured or intact C2C12 myotubes promote distinct responses converging toward myogenesis. Int. J. Mol. Sci. 18:2488. 10.3390/ijms18112488 29165341 PMC5713454

[B68] Guo B. B. Bellingham S. A. Hill A. F. (2015). The neutral sphingomyelinase pathway regulates packaging of the prion protein into exosomes. J. Biol. Chem. 290, 3455–3467. 10.1074/jbc.M114.605253 25505180 PMC4319014

[B69] Hanson P. I. Shim S. Merrill S. A. (2009). Cell biology of the ESCRT machinery. Curr. Opin. Cell Biol. 21, 568–574. 10.1016/j.ceb.2009.06.002 19560911 PMC6195420

[B70] Hemler M. E. (2003). Tetraspanin proteins mediate cellular penetration, invasion, and fusion events and define a novel type of membrane microdomain. Annu. Rev. Cell Dev. Biol. 19, 397–422. 10.1146/annurev.cellbio.19.111301.153609 14570575

[B71] Hendricks B. K. Shi R. (2014). Mechanisms of neuronal membrane sealing following mechanical trauma. Neurosci. Bull. 30, 627–644. 10.1007/s12264-013-1446-4 24993771 PMC5562621

[B72] Hindi S. M. Kumar A. (2016). Toll-like receptor signalling in regenerative myogenesis: friend and foe. J. Pathol. 239, 125–128. 10.1002/path.4714 26956975 PMC4957968

[B73] Hogarth M. W. Defour A. Lazarski C. Gallardo E. Manera J. D. Partridge T. A.. (2019). Fibroadipogenic progenitors are responsible for muscle loss in limb girdle muscular dystrophy 2B. Nat. Commun. 10:2430. 10.1038/s41467-019-10438-z 31160583 PMC6547715

[B74] Horak M. Novak J. Bienertova-Vasku J. (2016). Muscle-specific micrornas in skeletal muscle development. Dev. Biol. 10, 1–13. 10.1016/j.ydbio.2015.12.013 26708096

[B75] Horn A. Jaiswal J. K. (2018). Cellular mechanisms and signals that coordinate plasma membrane repair. Cell. Molecul. Life Sci. 75, 3751–3770. 10.1007/s00018-018-2888-7 30051163 PMC6541445

[B76] Horn A. Van Der Meulen J. H. Defour A. Hogarth M. Sreetama S. C. Reed A.. (2017). Mitochondrial redox signaling enables repair of injured skeletal muscle cells. Sci. Signal. 10:eaaj1978. 10.1126/scisignal.aaj1978 28874604 PMC5949579

[B77] Ieronimakis N. Pantoja M. Hays A. L. Dosey T. L. Qi J. Fischer K. A.. (2013). Increased sphingosine-1-phosphate improves muscle regeneration in acutely injured mdx mice. Skelet. Muscle 3:20. 10.1186/2044-5040-3-20 23915702 PMC3750760

[B78] Jaiswal J. K. Chakrabarti S. Andrews N. W. Simon S. M. (2004). Synaptotagmin VII restricts fusion pore expansion during lysosomal exocytosis. PLoS Biol. 2:E233. 10.1371/journal.pbio.0020233 15226824 PMC439782

[B79] Jaiswal J. K. Lauritzen S. P. Scheffer L. Sakaguchi M. Bunkenborg J. Simon S. M.. (2014). S100A11 is required for efficient plasma membrane repair and survival of invasive cancer cells. Nat. Commun. 5:3795. 10.1038/ncomms4795 24806074 PMC4026250

[B80] Janas T. Janas M. M. Sapon K. Janas T. (2015). Mechanisms of RNA loading into exosomes. FEBS Lett. 589, 1391–1398. 10.1016/j.febslet.2015.04.036 25937124

[B81] Janas T. Janas T. (2011). The selection of aptamers specific for membrane molecular targets. Cell Mol. Biol. Lett. 16, 25–39. 10.2478/s11658-010-0023-3 20585890 PMC6275783

[B82] Janas T. Janas T. Yarus M. (2012). Human tRNA(Sec) associates with HeLa membranes, cell lipid liposomes, and synthetic lipid bilayers. RNA 18, 2260–2268. 10.1261/rna.035352.112 23097422 PMC3504676

[B83] Jennings M. D. Pavitt G. D. (2010). eIF5 is a dual function GAP and GDI for eukaryotic translational control. Small GTPases 1, 118–123. 10.4161/sgtp.1.2.13783 21686265 PMC3116597

[B84] Jeppesen D. K. Fenix A. M. Franklin J. L. Higginbotham J. N. Zhang Q. Zimmerman L. J.. (2019). Reassessment of exosome composition. Cell 177, 428–445.e418. 10.1016/j.cell.2019.02.029 30951670 PMC6664447

[B85] Jimenez A. J. Maiuri P. Lafaurie-Janvore J. Divoux S. Piel M. Perez F. (2014). ESCRT machinery is required for plasma membrane repair. Science 343:1247136. 10.1126/science.1247136 24482116

[B86] Jin C. F. Li Y. Ding X. B. Li X. Zhang L. L. Liu X. F.. (2017). lnc133b, a novel, long non-coding rna, regulates bovine skeletal muscle satellite cell proliferation and differentiation by mediating mir-133b. Gene 630, 35–43. 10.1016/j.gene.2017.07.066 28757453

[B87] Juan A. H. Kumar R. M. Marx J. G. Young R. A. Sartorelli V. (2009). Mir-214-dependent regulation of the polycomb protein ezh2 in skeletal muscle and embryonic stem cells. Mol. Cell 361, 61–74. 10.1016/j.molcel.2009.08.008 PMC2761245 19818710

[B88] Jung H. L. Kwak H. E. Kim S. S. Kim Y. C. Lee C. D. Byurn H. K.. (2011). Effects of panax ginseng supplementation on muscle damage and inflammation after uphill treadmill running in humans. Am. J. Chin. Med. 39, 441–450. 10.1142/S0192415X11008944 21598413

[B89] Kajimoto T. Mohamed N. N. I. Badawy S. M. M. Matovelo S. A. Hirase M. Nakamura S.. (2018). Involvement of Gβγ subunits of Gi protein coupled with S1P receptor on multivesicular endosomes in F-actin formation and cargo sorting into exosomes. J. Biol. Chem. 293, 245–253. 10.1074/jbc.M117.808733 29133526 PMC5766922

[B90] Kajimoto T. Okada T. Miya S. Zhang L. Nakamura S. (2013). Ongoing activation of sphingosine 1-phosphate receptors mediates maturation of exosomal multivesicular endosomes. Nat. Commun. 4:2712. 10.1038/ncomms3712 24231649

[B91] Keyel P. A. Loultcheva L. Roth R. Salter R. D. Watkins S. C. Yokoyama W. M.. (2011). Streptolysin O clearance through sequestration into blebs that bud passively from the plasma membrane. J. Cell Sci. 124, 2414–2423. 10.1242/jcs.076182 21693578 PMC3124372

[B92] Kim H. K. Lee Y. S. Sivaprasad U. Malhotra A. Dutta A. (2006). Muscle-specific microrna mir-206 promotes muscle differentiation. J. Cell Biol. 174, 677–687. 10.1083/jcb.200603008 16923828 PMC2064311

[B93] Kim S. Lee M. J. Choi J. Y. Park D. H. Kwak H. B. Moon S.. (2018). Roles of exosome-like vesicles released from inflammatory C2C12 myotubes: regulation of myocyte differentiation and myokine expression. Cell Physiol. Biochem. 48, 1829–1842. 10.1159/000492505 30092568

[B94] Kowal J. Arras G. Colombo M. Jouve M. Morath J. P. Primdal-Bengtson B.. (2016). Proteomic comparison defines novel markers to characterize heterogeneous populations of extracellular vesicle subtypes. Proc. Natl. Acad. Sci. U.S.A. 113, E968–E977. 10.1073/pnas.1521230113 26858453 PMC4776515

[B95] Kowal J. Tkach M. Théry C. (2014). Biogenesis and secretion of exosomes. Curr. Opin. Cell Biol. 29, 116–125. 10.1016/j.ceb.2014.05.004 24959705

[B96] Lagrota-Candido J. Canella I. Pinheiro D. F. Santos-Silva L. P. Ferreira R. S. Guimaraes-Joca F. J.. (2010). Characteristic pattern of skeletal muscle remodelling in different mouse strains. Int. J. Exp. Pathol. 91, 522–529. 10.1111/j.1365-2613.2010.00737.x 20804543 PMC3010551

[B97] Lamanna F. Kirschbaum F. Waurick I. Dieterich C. Tiedemann R. (2015). Cross-tissue and cross-species analysis of gene expression in skeletal muscle and electric organ of african weakly-electric fish (Teleostei; Mormyridae). BMC Genomics 16:668. 10.1186/s12864-015-1858 26335922 PMC4558960

[B98] Laterza O. F. Lim L. Garrett-Engele P. W. Vlasakova K. Muniappa N. Tanaka W. K.. (2009). Plasma MicroRNAs as sensitive and specific biomarkers of tissue injury. Clin. Chem. 55, 1977–1983. 10.1373/clinchem.2009.131797 19745058

[B99] Le Bihan M. C. Bigot A. Jensen S. S. Dennis J. L. Rogowska-Wrzesinska A. Lainé J.. (2012). In-depth analysis of the secretome identifies three major independent secretory pathways in differentiating human myoblasts. J. Proteom. 77, 344–356. 10.1016/j.jprot.2012.09.008 23000592

[B100] Le Roy C. Wrana J. L. (2005). Clathrin- and non-clathrin-mediated endocytic regulation of cell signalling. Nat. Rev. Mol. Cell Biol. 6, 112–126. 10.1038/nrm1571 15687999

[B101] Leikina E. Defour A. Melikov K. Van Der Meulen J. H. Nagaraju K. Bhuvanendran S. (2015). Annexin A1 deficiency does not affect myofiber repair but delays regeneration of injured muscles. Sci. Rep. 5:18246 10.1038/srep18246 26667898 PMC4678367

[B102] Lemke G. (2019). How macrophages deal with death. Nat. Rev. Immunol. 10.1038/s41577-019-0167-y. [Epub ahead of print]. 31019284 PMC6733267

[B103] Leoni G. Alam A. Neumann P. A. Lambeth J. D. Cheng G. Mccoy J.. (2013). Annexin A1, formyl peptide receptor, and NOX1 orchestrate epithelial repair. J. Clin. Invest. 123, 443–454. 10.1172/JCI65831 23241962 PMC3533303

[B104] Leoni G. Neumann P. A. Kamaly N. Quiros M. Nishio H. Jones H. R.. (2015). Annexin A1-containing extracellular vesicles and polymeric nanoparticles promote epithelial wound repair. J. Clin. Invest. 125, 1215–1227. 10.1172/JCI76693 25664854 PMC4362251

[B105] Ling Y. H. Sui M. H. Zheng Q. Wang K. Y. Wu H. Li W. Y. (2018). mir-27b regulates myogenic proliferation and differentiation b targeting pax3 in goat. Sci. Rep. 8:3909 10.1038/s41598-018-22262-4 29500394 PMC5834623

[B106] Loh K. C. Leong W. I. Carlson M. E. Oskouian B. Kumar A. Fyrst H.. (2012). Sphingosine-1-phosphate enhances satellite cell activation in dystrophic muscles through a S1PR2/STAT3 signaling pathway. PLoS ONE 7:e37218. 10.1371/annotation/7e7ac57d-30ae-4e49-9138-e3bdbe3491d2 22606352 PMC3351440

[B107] Lopez-Verrilli M. A. Picou F. Court F. A. (2013). Schwann cell-derived exosomes enhance axonal regeneration in the peripheral nervous system. Glia 61, 1795–1806. 10.1002/glia.22558 24038411

[B108] Luchessi A. D. Cambiaghi T. D. Hirabara S. M. Lambertucci R. H. Silveira L. R. Baptista I. L.. (2009). Involvement of eukaryotic translation initiation factor 5A (eIF5A) in skeletal muscle stem cell differentiation. J. Cell Physiol. 218, 480–489. 10.1002/jcp.21619 19006180

[B109] Lv Z. Wei Y. Wang D. Zhang C. Y. Zen K. Li L. (2014). Argonaute 2 in cell-secreted microvesicles guides the function of secreted miRNAs in recipient cells. PLoS ONE 9:e103599. 10.1371/journal.pone.0103599 25072345 PMC4114802

[B110] Mancek-Keber M. Frank-Bertoncelj M. Hafner-Bratkovic I. Smole A. Zorko M. Pirher N.. (2015). Toll-like receptor 4 senses oxidative stress mediated by the oxidation of phospholipids in extracellular vesicles. Sci. Signal 8:ra60. 10.1126/scisignal.2005860 26082436

[B111] Mandato C. A. Bement W. M. (2001). Contraction and polymerization cooperate to assemble and close actomyosin rings around Xenopus oocyte wounds. J. Cell Biol. 154, 785–797. 10.1083/jcb.200103105 11502762 PMC2196444

[B112] Matsuzaka Y. Tanihata J. Komaki H. Ishiyama A. Oya Y. Rüegg U.. (2016). Characterization and functional analysis of extracellular vesicles and muscle-abundant miRNAs (miR-1, miR-133a, and miR-206) in C2C12 myocytes and mdx mice. PLoS ONE 11:e0167811. 10.1371/journal.pone.0167811 27977725 PMC5158003

[B113] Mayer U. (2003). Integrins: redundant or important players in skeletal muscle? J. Biol. Chem. 278, 14587–14590. 10.1074/jbc.R200022200 12556453

[B114] Mccarthy J. J. (2008). MicroRNA-206: the skeletal muscle-specific myomiR. Biochim. Biophys. Acta 1779, 682–691. 10.1016/j.bbagrm.2008.03.001 18381085 PMC2656394

[B115] Mcdade J. R. Archambeau A. Michele D. E. (2014). Rapid actin-cytoskeleton-dependent recruitment of plasma membrane-derived dysferlin at wounds is critical for muscle membrane repair. FASEB J. 28, 3660–3670. 10.1096/fj.14-250191 24784578 PMC4101652

[B116] Mcguinness D. Anthony D. F. Moulisova V. Macdonald A. I. Macintyre A. Thomson J. (2016). Microvesicles but not exosomes from pathfinder cells stimulate functional recovery of the pancreas in a mouse streptozotocin-induced diabetes model. Rejuvenat. Res. 19, 223–232. 10.1089/rej.2015.1723 26414011

[B117] Mcneil P. L. Clarke M. F. Miyake K. (2001). Cell wound assays. Curr. Protoc. Cell Biol. Chapter 12, Unit 12.4. 10.1002/0471143030.cb1204s02 18228318

[B118] Meldolesi J. (2018). Exosomes and ectosomes in intercellular communication. Curr. Biol. 28, R435–R444. 10.1016/j.cub.2018.01.059 29689228

[B119] Michailowsky V. Li H. Mittra B. Iyer S. R. Mazála D. A.G. Andrews N.W. (2019). Defects in sarcolemma repair and skeletal muscle function after injury in a mouse model of Niemann-Pick type A/B disease. Skelet. Muscle 9:1. 10.1186/s13395-018-0187-5 30611303 PMC6320626

[B120] Middel V. Zhou L. Takamiya M. Beil T. Shahid M. Roostalu U.. (2016). Dysferlin-mediated phosphatidylserine sorting engages macrophages in sarcolemma repair. Nat. Commun. 7:12875. 10.1038/ncomms12875 27641898 PMC5031802

[B121] Mulcahy L. A. Pink R. C. Carter D. R. (2014). Routes and mechanisms of extracellular vesicle uptake. J. Extracell. Vesicles 3. 10.3402/jev.v3.24641 25143819 PMC4122821

[B122] Muralidharan-Chari V. Clancy J. Plou C. Romao M. Chavrier P. Raposo G.. (2009). ARF6-regulated shedding of tumor cell-derived plasma membrane microvesicles. Curr. Biol. 19, 1875–1885. 10.1016/j.cub.2009.09.059 19896381 PMC3150487

[B123] Murphy C. Withrow J. Hunter M. Liu Y. Tang Y. L. Fulzele S.. (2018). Emerging role of extracellular vesicles in musculoskeletal diseases. Mol. Aspects Med. 60, 123–128. 10.1016/j.mam.2017.09.006 28965750 PMC5856577

[B124] Nabhan J. F. Hu R. Oh R. S. Cohen S. N. Lu Q. (2012). Formation and release of arrestin domain-containing protein 1-mediated microvesicles (ARMMs) at plasma membrane by recruitment of TSG101 protein. Proc. Natl. Acad. Sci. U.S.A. 109, 4146–4151. 10.1073/pnas.1200448109 22315426 PMC3306724

[B125] Nakamura Y. Miyaki S. Ishitobi H. Matsuyama S. Nakasa T. Kamei N.. (2015). Mesenchymal-stem-cell-derived exosomes accelerate skeletal muscle regeneration. FEBS Lett. 589, 1257–1265. 10.1016/j.febslet.2015.03.031 25862500

[B126] Nakazawa H. Yamada M. Tanaka T. Kramer J. Yu Y. M. Fischman A. J.. (2015). Role of protein farnesylation in burn-induced metabolic derangements and insulin resistance in mouse skeletal muscle. PLoS ONE 10:e0116633. 10.1371/journal.pone.0116633 25594415 PMC4296934

[B127] Nazarenko I. Rana S. Baumann A. Mcalear J. Hellwig A. Trendelenburg M.. (2010). Cell surface tetraspanin Tspan8 contributes to molecular pathways of exosome-induced endothelial cell activation. Cancer Res. 70, 1668–1678. 10.1158/0008-5472.CAN-09-2470 20124479

[B128] Ohtake Y. Tojo H. Seiki M. (2006). Multifunctional roles of MT1-MMP in myofiber formation and morphostatic maintenance of skeletal muscle. J. Cell Sci. 119(Pt 18), 3822–3832. 10.1242/jcs.03158 16926191

[B129] Oliveira G. P. Jr. Porto W. F. Palu C. C. Pereira L. M. Petriz B. Almeida J. A.. (2018). Effects of acute aerobic exercise on rats serum extracellular vesicles diameter, concentration and small RNAs content. Front. Physiol. 9:532. 10.3389/fphys.2018.00532 29881354 PMC5976735

[B130] Panagiotou N. Wayne Davies R. Selman C. Shiels P. G. (2016). Microvesicles as vehicles for tissue regeneration: changing of the guards. Curr. Pathobiol. Rep. 4, 181–187. 10.1007/s40139-016-0115-5 27882267 PMC5101251

[B131] Perez-Hernandez D. Gutiérrez-Vázquez C. Jorge I. López-Martín S. Ursa A. Sánchez-Madrid F.. (2013). The intracellular interactome of tetraspanin-enriched microdomains reveals their function as sorting machineries toward exosomes. J. Biol. Chem. 288, 11649–11661. 10.1074/jbc.M112.445304 23463506 PMC3636856

[B132] Phinney D. G. Di Giuseppe M. Njah J. Sala E. Shiva S. Croix C. M. St.. (2015). Mesenchymal stem cells use extracellular vesicles to outsource mitophagy and shuttle microRNAs. Nat. Commun. 6:8472. 10.1038/ncomms9472 26442449 PMC4598952

[B133] Redpath G. M. Woolger N. Piper A. K. Lemckert F. A. Lek A. Greer P. A.. (2014). Calpain cleavage within dysferlin exon 40a releases a synaptotagmin-like module for membrane repair. Mol. Biol. Cell 25, 3037–3048. 10.1091/mbc.e14-04-0947 25143396 PMC4230592

[B134] Rejman J. Oberle V. Zuhorn I. S. Hoekstra D. (2004). Size-dependent internalization of particles via the pathways of clathrin- and caveolae-mediated endocytosis. Biochem. J. 377, 159–169. 10.1042/bj20031253 14505488 PMC1223843

[B135] Roberts T. C. Blomberg K. E. Mcclorey G. El Andaloussi S. Godfrey C. Betts C.. (2012). Expression analysis in multiple muscle groups and serum reveals complexity in the microRNA transcriptome of the mdx mouse with implications for therapy. Mol. Ther. Nucleic Acids 1:e39. 10.1038/mtna.2012.26 23344181 PMC3437806

[B136] Romancino D. P. Paterniti G. Campos Y. De Luca A. Di Felice V. D'azzo A.. (2013). Identification and characterization of the nano-sized vesicles released by muscle cells. FEBS Lett. 587, 1379–1384. 10.1016/j.febslet.2013.03.012 23523921 PMC4714929

[B137] Romero M. Keyel M. Shi G. Bhattacharjee P. Roth R. Heuser J. E.. (2017). Intrinsic repair protects cells from pore-forming toxins by microvesicle shedding. Cell Death Differ. 24, 798–808. 10.1038/cdd.2017.11 28186501 PMC5423106

[B138] Roostalu U. Strähle U. (2012). *In vivo* imaging of molecular interactions at damaged sarcolemma. Dev. Cell 22, 515–529. 10.1016/j.devcel.2011.12.008 22421042

[B139] Saba J. D. De La Garza-Rodea A. S. (2013). S1P lyase in skeletal muscle regeneration and satellite cell activation: exposing the hidden lyase. Biochim. Biophys. Acta 1831, 167–175. 10.1016/j.bbalip.2012.06.009 22750505 PMC3609719

[B140] Sahoo S. Losordo D. W. (2014). Exosomes and cardiac repair after myocardial infarction. Circ. Res. 114, 333–344. 10.1161/CIRCRESAHA.114.300639 24436429

[B141] Sansone P. Savini C. Kurelac I. Chang Q. Amato L. B. Strillacci A.. (2017). Packaging and transfer of mitochondrial DNA via exosomes regulate escape from dormancy in hormonal therapy-resistant breast cancer. Proc. Natl. Acad. Sci. U.S.A. 114, E9066–E9075. 10.1073/pnas.1704862114 29073103 PMC5664494

[B142] Santangelo L. Giurato G. Cicchini C. Montaldo C. Mancone C. Tarallo R.. (2016). The RNA-binding protein SYNCRIP is a component of the hepatocyte exosomal machinery controlling MicroRNA sorting. Cell Rep. 17, 799–808. 10.1016/j.celrep.2016.09.031 27732855

[B143] Sassoli C. Formigli L. Bini F. Tani A. Squecco R. Battistini C.. (2011). Effects of S1P on skeletal muscle repair/regeneration during eccentric contraction. J. Cell Mol. Med. 15, 2498–2511. 10.1111/j.1582-4934.2010.01250.x 21199328 PMC3822960

[B144] Sawada S. Kon M. Wada S. Ushida T. Suzuki K. Akimoto T. (2013). Profiling of circulating micrornas after a bout of acute resistance exercise in humans. PLoS ONE 8:e70823. 10.1371/journal.pone.0070823 23923026 PMC3726615

[B145] Scheffer L. L. Sreetama S. C. Sharma N. Medikayala S. Brown K. J. Defour A.. (2014). Mechanism of Ca(2)(+)-triggered ESCRT assembly and regulation of cell membrane repair. Nat. Commun. 5:5646. 10.1038/ncomms6646 25534348 PMC4333728

[B146] Schneider D. J. Speth J. M. Penke L. R. Wettlaufer S. H. Swanson J. A. Peters-Golden M. (2017). Mechanisms and modulation of microvesicle uptake in a model of alveolar cell communication. J. Biol. Chem. 292, 20897–20910. 10.1074/jbc.M117.792416 29101235 PMC5743066

[B147] Shurtleff M. J. Temoche-Diaz M. M. Karfilis K. V. Ri S. Schekman R. (2016). Y-box protein 1 is required to sort microRNAs into exosomes in cells and in a cell-free reaction. Elife 5:e19276. 10.7554/eLife.19276 27559612 PMC5047747

[B148] Silva A. M. Teixeira J. H. Almeida M. I. Gonçalves R. M. Barbosa M. A. Santos S. G. (2017). Extracellular vesicles: immunomodulatory messengers in the context of tissue repair/regeneration. Eur. J. Pharm. Sci. 98, 86–95. 10.1016/j.ejps.2016.09.017 27644894

[B149] Simionescu-Bankston A. Leoni G. Wang Y. Pham P. P. Ramalingam A. Duhadaway J. B.. (2013). The N-BAR domain protein, Bin3, regulates Rac1- and Cdc42-dependent processes in myogenesis. Dev. Biol. 382, 160–171. 10.1016/j.ydbio.2013.07.004 23872330 PMC3783639

[B150] Siracusa J. Koulmann N. Bourdon S. Goriot M. E. Banzet S. (2016). Circulating miRNAs as biomarkers of acute muscle damage in rats. Am. J. Pathol. 186, 1313–1327. 10.1016/j.ajpath.2016.01.007 26952641

[B151] Small E. M. O'Rourke J. R. Moresi V. Sutherland L. B. McAnally J. Gerard R. D.. (2010). Regulation of pi3-kinase/akt signaling by muscle-enriched microrna-486. Proc. Natl. Acad. Sci. U.S.A. 107, 4218–4223. 10.1073/pnas.1000300107 20142475 PMC2840099

[B152] Spaeth C. S. Fan J. D. Spaeth E. B. Robison T. Wilcott R. W. Bittner G. D. (2012). Neurite transection produces cytosolic oxidation, which enhances plasmalemmal repair. J. Neurosci. Res. 90, 945–954. 10.1002/jnr.22823 22497022

[B153] Sreetama S. C. Takano T. Nedergaard M. Simon S. M. Jaiswal J. K. (2016). Injured astrocytes are repaired by Synaptotagmin XI-regulated lysosome exocytosis. Cell Death Differ. 23, 596–607. 10.1038/cdd.2015.124 26450452 PMC4986631

[B154] Statello L. Maugeri M. Garre E. Nawaz M. Wahlgren J. Papadimitriou A.. (2018). Identification of RNA-binding proteins in exosomes capable of interacting with different types of RNA: RBP-facilitated transport of RNAs into exosomes. PLoS ONE 13:e0195969. 10.1371/journal.pone.0195969 29689087 PMC5918169

[B155] Svensson K. J. Christianson H. C. Wittrup A. Bourseau-Guilmain E. Lindqvist E. Svensson L. M.. (2013). Exosome uptake depends on ERK1/2-heat shock protein 27 signaling and lipid Raft-mediated endocytosis negatively regulated by caveolin-1. J. Biol. Chem. 288, 17713–17724. 10.1074/jbc.M112.445403 23653359 PMC3682571

[B156] Swaggart K. A. Demonbreun A. R. Vo A. H. Swanson K. E. Kim E. Y. Fahrenbach J. P.. (2014). Annexin A6 modifies muscular dystrophy by mediating sarcolemmal repair. Proc. Natl. Acad. Sci. U.S.A. 111, 6004–6009. 10.1073/pnas.1324242111 24717843 PMC4000833

[B157] Tam C. Idone V. Devlin C. Fernandes M. C. Flannery A. He X.. (2010). Exocytosis of acid sphingomyelinase by wounded cells promotes endocytosis and plasma membrane repair. J. Cell Biol. 189, 1027–1038. 10.1083/jcb.201003053 20530211 PMC2886342

[B158] Taverna S. Pucci M. Alessandro R. (2017). Extracellular vesicles: small bricks for tissue repair/regeneration. Ann. Transl. Med. 5:83. 10.21037/atm.2017.01.53 28275628 PMC5337202

[B159] Teng Y. Ren Y. Hu X. Mu J. Samykutty A. Zhuang X.. (2017). MVP-mediated exosomal sorting of miR-193a promotes colon cancer progression. Nat. Commun. 8:14448. 10.1038/ncomms14448 28211508 PMC5321731

[B160] Tian T. Zhu Y. L. Zhou Y. Y. Liang G. F. Wang Y. Y. Hu F. H.. (2014). Exosome uptake through clathrin-mediated endocytosis and macropinocytosis and mediating miR-21 delivery. J. Biol. Chem. 289, 22258–22267. 10.1074/jbc.M114.588046 24951588 PMC4139237

[B161] Tidball J. G. (2011). Mechanisms of muscle injury, repair, and regeneration. Compr. Physiol. 1, 2029–2062. 10.1002/cphy.c100092 23733696

[B162] Tidball J. G. (2017). Regulation of muscle growth and regeneration by the immune system. Nat. Rev. Immunol. 17, 165–178. 10.1038/nri.2016.150 28163303 PMC5452982

[B163] Togo T. Krasieva T. B. Steinhardt R. A. (2000). A decrease in membrane tension precedes successful cell-membrane repair. Mol. Biol. Cell 11, 4339–4346. 10.1091/mbc.11.12.4339 11102527 PMC15076

[B164] Trajkovic K. Hsu C. Chiantia S. Rajendran L. Wenzel D. Wieland F.. (2008). Ceramide triggers budding of exosome vesicles into multivesicular endosomes. Science 319, 1244–1247. 10.1126/science.1153124 18309083

[B165] Vaittinen S. Lukka R. Sahlgren C. Hurme T. Rantanen J. Lendahl U.. (2001). The expression of intermediate filament protein nestin as related to vimentin and desmin in regenerating skeletal muscle. J. Neuropathol. Exp. Neurol. 60, 588–597. 10.1093/jnen/60.6.588 11398835

[B166] Vaughan E. M. You J. S. Elsie Yu H. Y. Lasek A. Vitale N. Hornberger T. A.. (2014). Lipid domain-dependent regulation of single-cell wound repair. Mol. Biol. Cell 25, 1867–1876. 10.1091/mbc.e14-03-0839 24790096 PMC4055266

[B167] Villarroya-Beltri C. Gutiérrez-Vázquez C. Sánchez-Cabo F. Pérez-Hernández D. Vázquez J. Martin-Cofreces N.. (2013). Sumoylated hnRNPA2B1 controls the sorting of miRNAs into exosomes through binding to specific motifs. Nat. Commun. 4:2980. 10.1038/ncomms3980 24356509 PMC3905700

[B168] Wang H. Wang B. (2016). Extracellular vesicle microRNAs mediate skeletal muscle myogenesis and disease. Biomed. Rep. 5, 296–300. 10.3892/br.2016.725 27588172 PMC4997983

[B169] Wang T. Gilkes D. M. Takano N. Xiang L. Luo W. Bishop C. J.. (2014). Hypoxia-inducible factors and RAB22A mediate formation of microvesicles that stimulate breast cancer invasion and metastasis. Proc. Natl. Acad. Sci. U.S.A. 111, E3234–3242. 10.1073/pnas.1410041111 24938788 PMC4128139

[B170] Whitham M. Parker B. L. Friedrichsen M. Hingst J. R. Hjorth M. Hughes W. E.. (2018). Extracellular vesicles provide a means for tissue crosstalk during exercise. Cell Metab. 27, 237–251.e234. 10.1016/j.cmet.2017.12.001 29320704

[B171] Willms E. Johansson H. J. Mäger I. Lee Y. Blomberg K. E. Sadik M.. (2016). Cells release subpopulations of exosomes with distinct molecular and biological properties. Sci. Rep. 6:22519. 10.1038/srep22519 26931825 PMC4773763

[B172] Windbanks C. E. Beyer C. Hagg A. Qiab H. Sepulveda P. V. Gregorevic P. (2013). mir-206 represses hypertrophy of myogenic cells but not muscle fibers via inhibition of hdac4. PLoS ONE 8:e73589 10.1371/journal.pone.0073589 24023888 PMC3759420

[B173] Wosczyna M. N. Rando T. A. (2018). A muscle stem cell support group: coordinated cellular responses in muscle regeneration. Dev. Cell 46, 135–143. 10.1016/j.devcel.2018.06.018 30016618 PMC6075730

[B174] Yáñez-Mó M. Barreiro O. Gordon-Alonso M. Sala-Valdés M. Sánchez-Madrid F. (2009). Tetraspanin-enriched microdomains: a functional unit in cell plasma membranes. Trends Cell Biol. 19, 434–446. 10.1016/j.tcb.2009.06.004 19709882

[B175] Yáñez-Mó M. Siljander P. R. Andreu Z. Zavec A. B. Borràs F. E. Buzas E. I.. (2015). Biological properties of extracellular vesicles and their physiological functions. J. Extracell Vesicles 4:27066. 10.3402/jev.v4.27066 25979354 PMC4433489

[B176] Yang J. M. Gould S. J. (2013). The cis-acting signals that target proteins to exosomes and microvesicles. Biochem. Soc. Trans. 41, 277–282. 10.1042/BST20120275 23356297

[B177] Yin H. Price F. Rudnicki M. A. (2013). Satellite cells and the muscle stem cell niche. Physiol. Rev. 93, 23–67. 10.1152/physrev.00043.2011 23303905 PMC4073943

[B178] Yuan J. Liu H. Gao W. Zhang L. Ye Y. Yuan L.. (2018). MicroRNA-378 suppresses myocardial fibrosis through a paracrine mechanism at the early stage of cardiac hypertrophy following mechanical stress. Theranostics 8, 2565–2582. 10.7150/thno.22878 29721099 PMC5928909

[B179] Yuasa K. Hagiwara Y. Ando M. Nakamura A. Takeda S. Hijikata T. (2008). MicroRNA-206 is highly expressed in newly formed muscle fibers: implications regarding potential for muscle regeneration and maturation in muscular dystrophy. Cell Struct. Funct. 33, 163–169. 10.1247/csf.08022 18827405

[B180] Zanotti S. Gibertini S. Blasevich F. Bragato C. Ruggieri A. Saredi S.. (2018). Exosomes and exosomal miRNAs from muscle-derived fibroblasts promote skeletal muscle fibrosis. Matrix Biol. 74, 77–100. 10.1016/j.matbio.2018.07.003 29981373

[B181] Zhang G. Liu Z. Ding H. Zhou Y. Doan H. A. Sin K. W. T.. (2017). Tumor induces muscle wasting in mice through releasing extracellular hsp70 and hsp90. Nat. Commun. 8:589. 10.1038/s41467-017-00726-x 28928431 PMC5605540

[B182] Zhang M. M. Hang H. C. (2017). Protein S-palmitoylation in cellular differentiation. Biochem. Soc. Trans. 45, 275–285. 10.1042/BST20160236 28202682 PMC5310721

[B183] Zhou T. Prather E. R. Garrison D. E. Zuo L. (2018). Interplay between ROS and antioxidants during ischemia-reperfusion injuries in cardiac and skeletal muscle. Int. J. Mol. Sci. 19:417. 10.3390/ijms19020417 29385043 PMC5855639

